# CHIMs are versatile cholesterol analogs mimicking and visualizing cholesterol behavior in lipid bilayers and cells

**DOI:** 10.1038/s42003-021-02252-5

**Published:** 2021-06-11

**Authors:** Anna L. L. Matos, Fabian Keller, Tristan Wegner, Carla Elizabeth Cadena del Castillo, David Grill, Sergej Kudruk, Anne Spang, Frank Glorius, Andreas Heuer, Volker Gerke

**Affiliations:** 1 Institute of Medical Biochemistry, Center for Molecular Biology of Inflammation, University of Münster, Münster, Germany; 2 Physical Chemistry Institute, University of Münster, Münster, Germany; 3 Center for Multiscale Theory and Computation (CMTC), Münster, Germany; 4 Institute of Organic Chemistry, University of Münster, Münster, Germany; 5 Biozentrum, University of Basel, Basel, Switzerland

**Keywords:** Computational chemistry, Cell biology

## Abstract

Cholesterol is an essential component of cellular membranes regulating the structural integrity and fluidity of biological bilayers and cellular processes such as signal transduction and membrane trafficking. However, tools to investigate the role and dynamics of cholesterol in live cells are still scarce and often show limited applicability. To address this, we previously developed a class of imidazolium-based cholesterol analogs, CHIMs. Here we confirm that CHIM membrane integration characteristics largely mimic those of cholesterol. Computational studies in simulated phospholipid bilayers and biophysical analyses of model membranes reveal that in biologically relevant systems CHIMs behave similarly to natural cholesterol. Importantly, the analogs can functionally replace cholesterol in membranes, can be readily labeled by click chemistry and follow trafficking pathways of cholesterol in live cells. Thus, CHIMs represent chemically versatile cholesterol analogs that can serve as a flexible toolbox to study cholesterol behavior and function in live cells and organisms.

## Abstract (web summary)

Matos et al characterise previously developed cholesterol analogues, named CHIMs, which can be fluorescently labelled by click-chemistry. They find that CHIMs segregate similarly to cholesterol in lipid bilayers, allow the visualisation of cholesterol dynamics in cells, and can functionally replace cholesterol in *C.elegans*.

## Introduction

Biological membranes are composed of different types of lipids, phospholipids, sphingolipids and cholesterol, and integral as well as associated proteins. They can adopt a vast array of configurations and are highly dynamic in nature, allowing rapid lateral diffusion and thus assembly and disassembly of their principal components. This activity enables membranes to fulfill highly versatile biological functions that range from constituting a mere scaffold and diffusion barrier to forming a two-dimensional platform for biochemical reactions.

Cholesterol is an important component of biological membranes. It regulates the cell membrane’s structural integrity and fluidity and plays a crucial role in mediating signal transduction and membrane trafficking processes^1–4^. However, the exact mode of action of cholesterol in these events is still not fully understood and tools to study cholesterol-mediated processes in an unperturbed biological environment are still limited and often suffer from major disadvantages^5,6^. Of prime importance for accessing cholesterol’s cellular functions is the possibility to visualize its subcellular distribution with high spatiotemporal resolution, for instance, by employing fluorescence microscopy techniques. As cholesterol does not show intrinsic fluorescence, modifications of the sterol backbone introducing a fluorescent moiety or fluorescent cholesterol-sensing molecules were developed for this purpose. These include cholesterol-binding agents such as filipin and perfringolysin O, chemically related steroids such as dehydroergosterol, which are intrinsically fluorescent, and fluorescently labeled cholesterol derivatives, mostly either modified at the C3-position (3-NBD-Cholesterol), at the double bond (6-Dansyl-Cholestanol), or at the terminus of the alkyl backbone (TopFluor/BODIPY-cholesterol). However, the dynamic visualization of cholesterol distribution in live cells using these markers has proven to be difficult, e.g., due to poor delivery of the labeled derivatives into cells, the prerequisite for cell fixation, relatively weak staining and resolution, or perturbation of the native membrane organization because of less efficient packing^7,8^.

**Fig 1:**
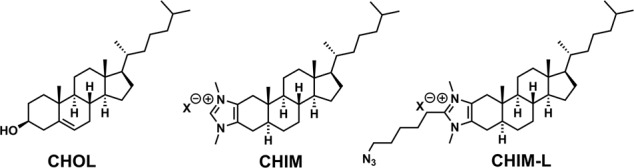
Imidazolium-based cholesterol analogs CHIM and CHIM-L. Molecular structures of natural cholesterol (CHOL) and the herein characterized CHIM analogs (X = Cl, I).

We therefore developed a class of imidazolium-based lipids including cholesterol analogs that integrate into model and cellular membranes^9–14^. These include cholesterol analogs (Fig. 1), herein referred to as CHIM (for CHolesterol-based IMidazolium salt), in which the hydroxyl group of cholesterol was converted into a polar, positively charged imidazolium moiety that enables flexible modifications while preserving cholesterol’s natural amphiphilic character. Among other things, CHIMs can be modified at the C2 position in a highly versatile manner, e.g., by introducing an azide linker to which a fluorophore label can be attached by SPAAC click chemistry^15^ and which we will refer to as CHIM-L throughout the paper. Importantly, the directionality of the imidazolium moiety leads to a positioning of the attached fluorophore outside of the membrane and therefore is predicted to not affect the proper membrane integration of the cholesterol backbone. Due to its unique design CHIM addresses many drawbacks of established cholesterol analogs; it should enable the visualization of cholesterol distribution in live cells without the need for cell fixation and should allow a flexible on-demand attachment of any fluorophore of choice or other functional groups (e.g. photoswitches, crosslinkers, or biotin labels) that could be used for the investigation and manipulation of the native cholesterol membrane environment. Our comprehensive characterization shows that CHIMs mimic cholesterol behavior in cellular membranes and can functionally replace endogenous cholesterol in the model organism *C. elegans*. Furthermore, combined computational, biophysical, and biochemical analyses reveal that membrane integration and dynamics of the chemically versatile CHIM derivatives can be controlled by chemical modification and membrane composition. Therefore, we envision that this highly modifiable class of cholesterol analogs will serve as important tools and flexible platforms for the investigation of cholesterol-dependent membrane processes.

## Results

### Characterization of CHIM within different bilayer environments via extensive molecular dynamics simulations

**Fig 2:**
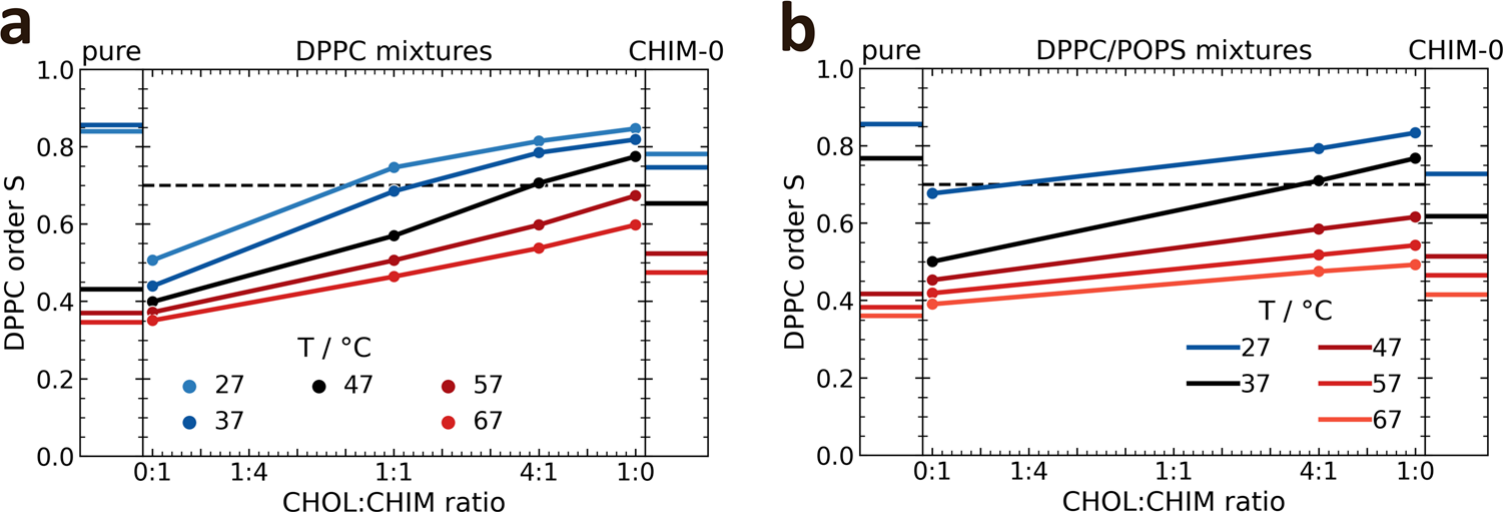
Effect of cholesterol and CHIM on order parameters in DPPC bilayers. Order of DPPC chains in DPPC (**a**) and DPPC/POPS (2:1) (**b**) mixtures containing CHOL, CHIM or a mixture of CHIM/CHOL, respectively, as a function of the cholesterol fraction within a total sterol concentration of 30 (**a**) and 20 (**b**) mol%. For comparison, the order of a pure DPPC bilayer and respective bilayers containing only CHIM-0 (the arbitrary charge-neutral CHIM-analog) is shown in the left and right panels, respectively. The color codes indicate the MD simulation temperatures, separating them in regimes of below (blues), above (reds), and near phase transition (black). The black dashed line is included to better distinguish between an ordered (S > 0.7) and disordered (S < 0.7) phase.

One of cholesterol’s most prominent characteristic is its ability to order alkyl chains of glycerol- and sphingolipids, thereby regulating the fluidity and packing density of membranes. To investigate whether CHIM shares this fundamental property of natural cholesterol we analyzed its ordering abilities in molecular dynamics (MD) simulations by calculating the median of the DPPC chain order parameter in DPPC/sterol bilayers, containing 30 mol% of CHIM, cholesterol (CHOL) or a mix of both with ratios of 1:4 and 1:1 CHIM to CHOL, respectively (Fig. 2a). In the MD simulations we focused on a comparison between cholesterol and CHIM to characterize the effect of changing the hydroxyl group to an imidazolium salt residue. The linker introduced in CHIM-L, the derivative used for cell labeling experiments (see below), is unlikely to affect the membrane integration properties of CHIM in a significant manner as revealed by analyzing a slightly modified CHIM-L (Supplementary Note and Supplementary Fig. 1). The amount of 30 mol% sterol was chosen to have a strong impact of these moieties on bilayer properties and because it resembles the amount of CHOL found in natural plasma membranes (PMs)^16,17^. To analyze the phase behavior, we simulated temperatures from 20 °C above to 20 °C below phase transition of the pure DPPC bilayer system (Fig. 2a, left panel); based on the nonlinear increase of average order parameters of the DPPC/CHOL mixtures at roughly S = 0.7, bilayers with an average order parameter of S > 0.7 were defined as ordered, otherwise as disordered. Based on the phase behavior of pure DPPC bilayers within the simulations, the data can be separated into two distinct temperature regimes: a high-temperature regime (reds, T > 47 °C) and a low-temperature regime (blues, T < 47 °C) with the black curve showing simulations at 47 °C (Fig. 2a, left panel). In the low-temperature regime and at CHIM/CHOL ratios of 1:1 or lower, the presence of CHIM only slightly affected the bilayer structure, while exceeding amounts of CHIM precluded the transition to an ordered phase. In the high-temperature regime, the DPPC bilayers containing only CHIM exhibited the same order as the pure DPPC bilayer. The linear dependence of the order parameter on the CHOL fraction suggests that in these conditions CHIM does not act as CHOL with respect to generating order in the DPPC bilayer.

CHIM’s distinct behavior within the two regimes may be related to its position within the bilayer. In DPPC/CHIM mixtures, the sterol ring of CHIM was found to be closer to the bilayer headgroup region than that of cholesterol (Supplementary Fig. 2). This position, derived from the density profiles of reference atoms (phosphor of phospholipids and C3 carbon of cholesterol and analogs) (Supplementary Fig. 3), was significantly changed when cholesterol was introduced and, already in a 1:1 CHIM/CHOL mixture, CHIM was tucked towards the acyl chain region. In contrast, natural cholesterol’s position remained unaffected up to a 1:1 CHIM/CHOL ratio. The alignment of CHIM’s sterol ring position with that of cholesterol’s sterol ring when mixing both sterols connotes CHIM’s similarity to CHOL in such an environment.

### CHIM’s ordering capabilities in lipid bilayers are mainly affected by its charge

The charge of CHIM is one significant difference to the structure of natural cholesterol and we hypothesized that it presumably is dominant in the above-described effects on order. At low concentrations, these charge effects are likely without relevance and thus CHIM behaves similar to cholesterol in these conditions. To examine potential charge effects, we performed simulations of the same binary mixtures (DPPC/CHIM) with an artificially charge-neutralized moiety termed CHIM-0 (Supplementary Fig. 4). The ordering capability of CHIM-0 was found to be far stronger than that of CHIM (Fig. 2a, right panel). In the low-temperature regime, the formation of an ordered phase was preserved, whereas in the high-temperature regime the ordering capability was approximately half that of CHOL. The finding that uncharged CHIM-0 maintained CHOL’s properties with respect to the overall phase behavior of DPPC bilayers is supported by the observation that CHOL and CHIM-0 share similar positions within bilayers of DPPC (Supplementary Fig. 2).

In native PMs negatively charged lipids comprise a small but significant proportion of the overall lipids. As these could affect CHIM’s properties by shielding its positive charge in the head region, we also simulated CHIM’s membrane integration in bilayers containing the negatively charged phospholipid POPS and employed phospholipid (PL) mixtures of DPPC/POPS with 20% of either CHIM, CHOL or mixtures of CHIM/CHOL (Fig. 2b). The impact of CHIM-0 on the DPPC order in DPPC/POPS/CHIM-0 bilayers was similar to that observed in the respective DPPC/CHIM-0 bilayers indicating that the presence of POPS had basically no impact on CHIM-0 properties (see right parts of panels a and b in Fig. 2). In the case of DPPC/POPS/CHIM bilayers, however, POPS indeed showed an effect, resulting in CHIM’s properties to become more closely related to those of natural cholesterol. Unlike in the DPPC bilayers without POPS, CHIM does not disrupt the order in the low-temperature regime and even pure CHIM had an ordering effect in the high-temperature regime, indicating that CHIM has ordering capabilities itself, albeit somewhat weaker than CHOL. Since the DPPC/POPS bilayer containing only CHIM behaves very similar to that containing only CHIM-0, the impact of CHIM’s charge appears to be largely compensated by POPS. Moreover, the behavior of the 1:4 system linearly interpolates between the pure CHIM and pure CHOL systems, suggesting that the CHOL-like properties of CHIM are intrinsic and not modified by the simultaneous presence of CHOL. Finally, the position of CHIM in the DPPC/POPS bilayers was similar to the position in the bilayers without POPS (Supplementary Fig. 2). Thus, POPS seems to be able to stabilize the headgroup region of CHIM in phospholipid bilayers containing higher concentrations of CHIM.

### CHIM shows segregation behavior similar to that of cholesterol in DPPC:DLiPC bilayers

**Fig 3:**
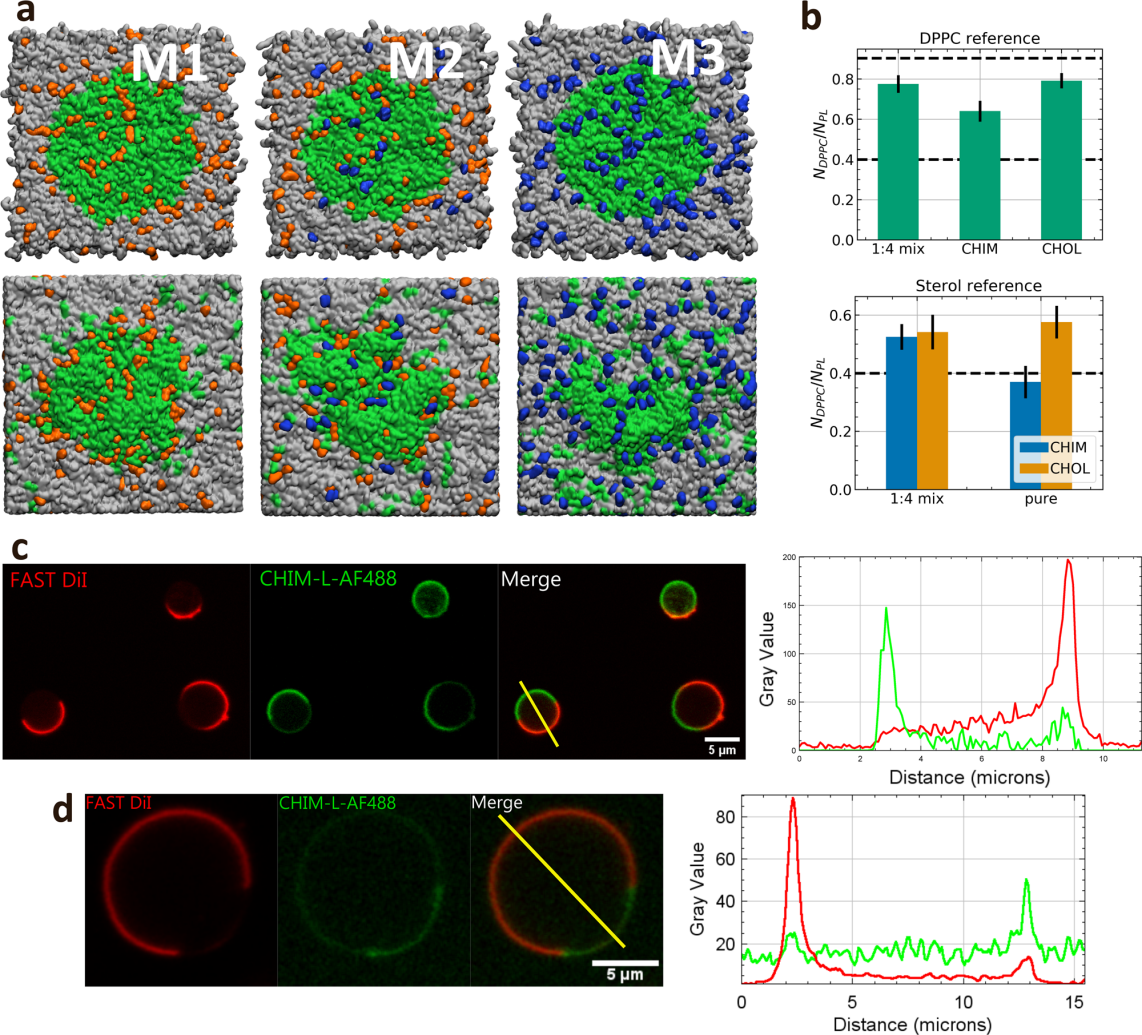
Lipid segregation in cholesterol and CHIM containing membranes. **a** Top view of a DPPC:DLiPC:sterol bilayer. DPPC is shown in green, DLiPC in gray, CHOL in orange, and CHIM in blue. The top row shows snapshots of the initial configuration while the bottom row shows snapshots of the final configuration after a simulation of at least 3 µs. **b** Top: Average ratio of DPPC neighbors over the total number of PL neighbors around DPPC averaged over the last 25% of the simulation. Bottom: Average ratio of DPPC neighbors over total PL neighbors around CHOL or CHIM. The dashed lines at 0.9 indicate complete segregation and at 0.4 complete mixing. **c** GUVs composed of POPS:DLiPC:DPPC:CHIM-L (14.29:42.85:28.57:14.29), stained with FAST DiI and clicked with AF488. Lipid segregation occurs at RT, as revealed by FAST DiI labeling of the Ld phase. Note that CHIM-L (labeled by the click reaction with AF488) shows a tendency to partition into Lo, as also seen in the line plot on the right. **d** DCA-treated GPMVs derived from HUVEC labeled with FAST DiI and fluorescently labeled CHIM-L (AF488). Note that CHIM partitions into the more ordered phase (Lo) negative for FAST DiI. The corresponding line plot (fluorescence intensity) for the vesicle is displayed on the right.

Next, we investigated CHIM’s membrane phase segregation behavior in comparison to that of natural cholesterol, which preferentially assembles into liquid-ordered domains together with saturated phospholipids. Therefore, we first simulated the dynamic behavior of a preformed DPPC patch in a DPPC:DLiPC bilayer without sterol molecules. We recorded snapshots of the initial and final configurations at different temperatures after simulation times of at least 500 ns and determined the fraction of DPPC neighbors overall PL neighbors as a function of simulation time (Supplementary Fig. 5a and 5b). Fractions of 0.9 indicate complete segregation of DPPC and DLiPC, while fractions of 0.4 indicate random mixing for this setup of a 4:6 DLiPC:DPPC ratio. At temperatures above 47 °C, complete and rapid mixing of the two lipid species was observed, whereas at 27 °C the DPPC patch was stable with the fraction of DPPC neighbors remaining at roughly 0.8 (segregation) even after 1.5 µs simulation time. At 37 °C the DPPC neighbor fraction slowly decreased to roughly 0.6, indicative of a slow mixing process. Due to the overall patch stability, a temperature of 27 °C was chosen to further investigate the impact of CHIM and/or CHOL on the segregation behavior. Specifically, we compared three different sets of randomly distributed sterols: pure CHOL (M1), pure CHIM (M3), and a 1:4 mixture of CHIM/CHOL (M2) with each bilayer containing a total of 16 mol% sterols (Fig. 3a). Already a qualitative evaluation of the lipid segregation behavior revealed different characteristics within the three sets. A strong deformation of the initial DPPC patch was observed for the pure CHIM system (M3), while the DPPC patch in the pure CHOL systems (M1) remained stable. Moreover, in bilayer M1, most cholesterol molecules were located within or near the DPPC patch, while in M3, the CHIM molecules were mostly statistically distributed within the bilayer. In the membrane simulation of a CHIM/CHOL mixture (M2), the DPPC patch, though slightly more deformed than in the pure CHOL system, remained intact and segregation between DPPC and DLiPC was preserved.

The quantification of the DPPC lipid environment in such bilayers revealed a change of CHIM’s segregation behavior depending on the CHIM/CHOL ratio (Fig. 3b, top). A slight mixing starting from complete segregation with DPPC neighbor ratios of 0.9 (dashed line) was visible in all simulation setups. In the case of pure CHIM, the relative number dropped to 0.65, indicating an ongoing mixing of DPPC and DLiPC, while in M1 and M2, the relative number ended at appr. 0.8. This shows that a modest presence of CHIM does not alter the stability of the DPPC patch. In the mixed system the DPPC enrichment is the same around CHIM and CHOL being in both cases significantly above random mixing and, within the margin of error, similar to the case of pure CHOL (Fig. 3b, bottom). In contrast, for the pure CHIM system a random distribution is observed.

Thus, in agreement with the analysis of the order parameters, even in the case of uncharged PL bilayers CHIM behaves very similar to natural cholesterol in ordered regimes (i.e., in DPPC rich regimes at low temperatures) as long as it is the minority component as compared to natural cholesterol.

### The partitioning of CHIM-L in model membranes mimics that of cholesterol as revealed by fluorescence microscopy and lipid–protein interaction studies

Based on the thorough computational characterization, we next investigated experimentally to what extent CHIM exhibits cholesterol-like behavior in biological membranes. First, the phase partitioning of CHIM was analyzed in both, artificial and cell-derived model membranes. In these and the following experiments we chose CHIM-L as derivative because it could be labeled with fluorescent dyes by Cu-free click chemistry at a site residing outside of the bilayer (Fig. 1). On the one hand, we employed giant unilamellar vesicles (GUVs) with known and adjustable lipid composition, and on the other hand, giant plasma membrane vesicles (GPMVs) resembling the lipid composition of the cellular plasma membrane (PM). The presence of fluorescently labeled CHIM-L precluded the formation of GUVs with the simple non-charged lipid composition DLiPC:DPPC:CHIM-L (60:40:20), likely due to CHIM-L’s positive charge. Addition of POPS, yielding a GUV composition of POPS:DLiPC:DPPC:CHIM-L (14.29:42.85:28.57:14.29), led to a compensation of the positive charge of CHIM-L and the formation of proper GUVs. In these GUVs, CHIM-L exhibited a tendency to segregate into liquid-ordered (Lo) domains at room temperature and avoided the more disordered domains labeled by FAST DiI (Fig. 3c). This is in line with the above MD simulations. GPMVs derived from primary human endothelial cells (HUVECs) were employed as a model membrane system with plasma membrane-like characteristics, including a negative surface charge, which was shown by the MD simulations to abrogate the packing order perturbation displayed by the positively charged CHIM. Lipid phase segregation in these GPMVs can be induced at room temperature by deoxycholic acid (DCA) treatment and then be visualized by staining with FAST DiI^18^. When GPMVs were prepared from cells treated with CHIM-L, which was subsequently labeled by click reaction with DBCO Alexa Fluor 488 (AF488), DCA treatment resulted in a separation of the FAST DiI-labeled Ld phase from the Lo membrane domain containing CHIM-AF488 (Fig. 3d). Together, these data indicate that CHIM-L mimics the partitioning behavior of cholesterol in GUV and GPMV membrane systems.

Cholesterol-like properties of CHIM-L were also analyzed by assessing protein-membrane interactions known to be affected by cholesterol. Here, we focused on the peripheral phospholipid-binding protein annexin A2 (AnxA2), which interacts with membranes in a cooperative manner in the presence but not absence of cholesterol^19,20^. As revealed by QCM-D (Quartz Crystal Microbalance with Dissipation) measurements employing bilayers containing negatively charged phospholipids, CHIM could faithfully replace cholesterol in triggering cooperativity in the AnxA2-membrane interaction (Supplementary Fig. 6). The nonlinear regression of the respective binding isotherms as a function of AnxA2 concentration showed a sigmoidal shape typical for positive cooperativity (n_Hill_ = 1.88 ± 0.13, R^2^ = 0.98). This cooperative binding, most likely reflecting a rearrangement of lipids upon interaction with AnxA2^21^, is not seen when the same bilayer devoid of CHIM or cholesterol is employed in the binding experiments^19^.

### CHIM is incorporated into cellular membranes and can visualize the dynamic distribution of cholesterol

**Fig 4:**
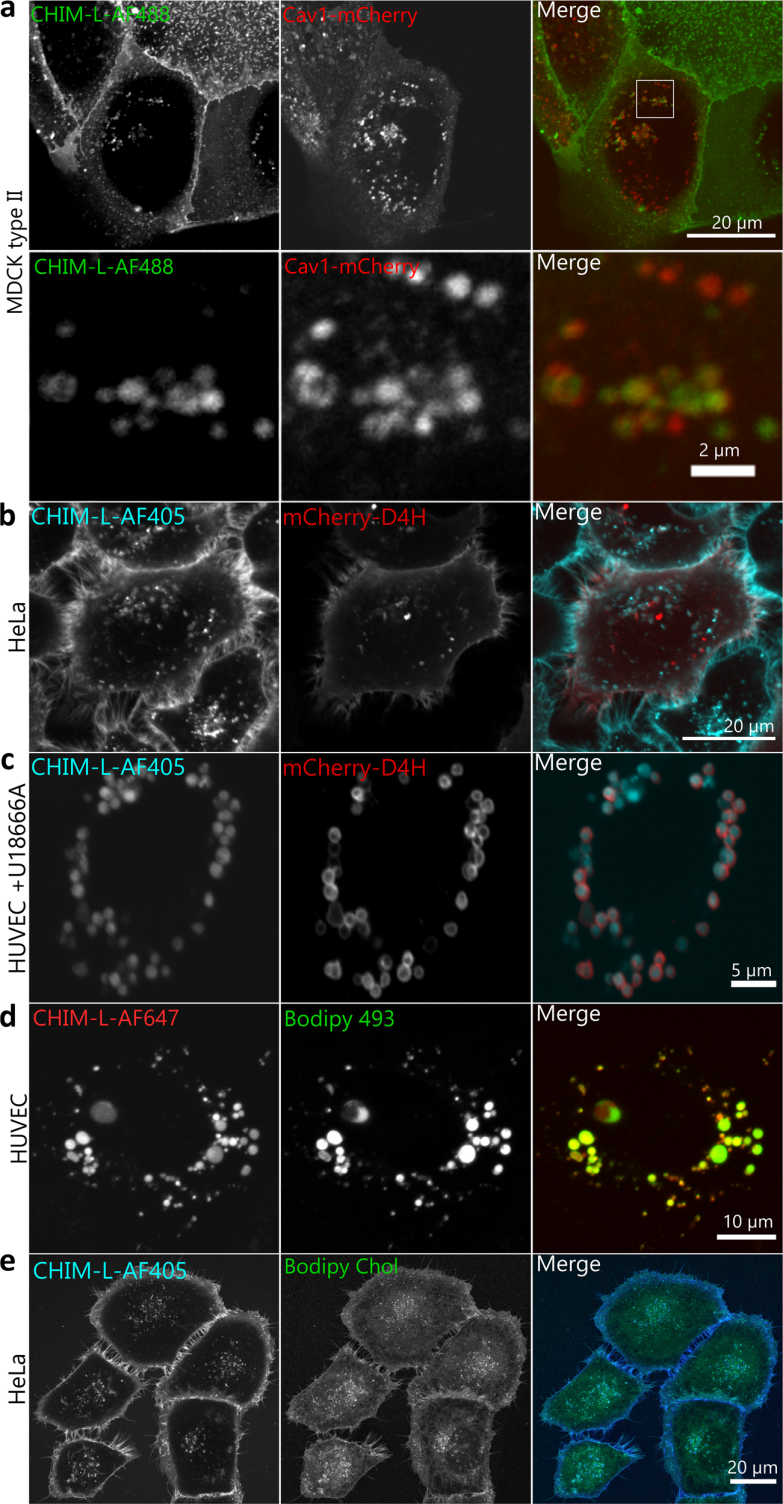
CHIM-L mimics the cholesterol distribution in live cells. Stills of live cell microscopy recordings revealing the CHIM-L distribution in epithelial cell lines and primary endothelial cells in comparison to different markers. **a** Caveolin1 (mCherry) enriched structures partially colocalize with fluorescently labeled CHIM-L (AF488) in MDCK cells. The inset of the merged panel is shown at higher maginfication in the bottom row. **b** CHIM-L (AF405) colocalizes with the ectopically expressed cholesterol-binding probe mCherry-D4H at the plasma membrane and some membrane invaginations/internal structures in HeLa cells. **c** Treatment with the NPC1 inhibitor U18666A, which results in cholesterol accumulation in enlarged late endosomes, sequesters CHIM-L (AF405) into these late endosomes that are also stained with mCherry-D4H in primary endothelial cells (HUVEC). **d** Lipid droplets stained with Bodipy 493 in comparison to CHIM-L (AF647) in HUVEC. **e** HeLa cells co-incubated with CHIM-L (AF405) and BODIPY-cholesterol as described in Materials and Methods. Note that CHIM-L shows a more profound plasma membrane staining.

As CHIM-L behaves similarly to cholesterol in model membranes, we next tested whether this is also observed in the membrane system of live cells. We first concentrated on caveolae, which represent cholesterol-rich PM invaginations functioning for instance as mechanosensors and cellular signaling regulators^22,23^. Cholesterol is recruited to such raft-like areas by interaction with the prime protein constituent caveolin1 (Cav1)^23^ and serves a structural role as removal of cholesterol causes disruption and flattening of caveolae^24,25^. As the levels of cholesterol or sphingolipids rise, caveolae are internalized, indicating that caveolae can sense the PM lipid composition^26^. To assess whether CHIM-L shows an enrichment in PM caveolae that is typical for endogenous cholesterol, we chose MDCK (Madin–Darby canine kidney) type II cells, a well-established model known to possess abundant caveolae^27^. We found that fluorescently labeled CHIM-L added to the culture medium was readily integrated into the PM and enriched in regions partially also positive for Cav1-mCherry (Fig. 4a). CHIM-L’s preferential PM association could also be visualized by ectopic intracellular expression of the cholesterol-binding probe D4H that preferentially labels the cholesterol-rich PM (Fig. 4b)^28^. Upon internalization of caveolae and other cholesterol-rich PM structures, natural cholesterol is delivered to the cell’s endosomal system (Supplementary Fig. 7). To address whether CHIM-L follows the same internalization route, we used markers for early and late endosomes, Rab5 and Rab7, and analyzed the intracellular distribution of fluorescently labeled CHIM-L, observed after longer incubation times, in comparison to these markers. To extend our analysis to primary cells, intracellular trafficking studies were carried out in endothelial cells isolated from umbilical cord veins (HUVEC). We found that internalized CHIM-L colocalizes with both endosomal markers (Supplementary Figs. 8 and 9). Moreover, this colocalization followed internalization kinetics, which are in line with a trafficking of CHIM-L through first early (Rab5) and then late endosomes (Rab7)^29^. Interestingly, the arrangement of CHIM-L in the endocytic organelles appeared to change as it was present in the limiting membrane of early endosomes but seemed to concentrate in the internal lumen of late endosomes. The accumulation of CHIM-L in the lumen of late endosomes/lysosomes was seen even more pronounced when cholesterol egress from these organelles was blocked by the drug U18666A, which inhibits the cholesterol transport protein Niemann Pick type C1 (NPC1) (Fig. 4c and Supplementary Fig. 10). This again reflected the behavior of natural cholesterol.

Cholesterol and caveolar components, in particular Cav1, also associate with lipid droplets (LDs), cellular reservoirs of fatty acids and cholesterol that are characterized by a hydrophobic core containing neutral lipids and cholesterol esters and a surrounding phospholipid monolayer^30^. Interestingly, internalized CHIM-L also colocalized with the LD marker Bodipy 493 (Fig. 4d and Supplementary Fig. 11) suggesting that CHIM-L, as a cholesterol analog that cannot be esterified, is also transported to LDs. We also compared cell labeling by CHIM-L to that of other labeled cholesterol derivative, BODIPY-cholesterol, which has often been used in such type of experiments. BODPIY-cholesterol when given for 60 min at room temperature, also stained the plasma membrane of HeLa cells albeit less prominently than AF405-labeled CHIM-L (Fig. 4e). Moreover, BODIPY-cholesterol, again similar to natural cholesterol and CHIM-L, accumulated in the lumen of late endosomes/lysosomes when cells were treated overnight with the drug U18666A (Supplementary Fig. 10). Various reports have also shown that BODIPY-cholesterol exhibits cholesterol-like properties in model membranes such as GPMVs^5^, thus extending the similarity to CHIM-L. However, it should be noted that BODIPY-cholesterol shows a lower degree of plasma membrane staining and a more pronounced intracellular distribution as compared to CHIM-L (Fig. 4e), possibly due to the fact that the BODIPY label is located in the acyl chain (i.e., inside the bilayer) and thereby precludes a very efficient membrane incorporation. In the CHIM-L, on the other hand, the fluorescent dye is attached outside of the membrane and thus most likely does not interfere with a proper bilayer incorporation of the sterol backbone.

Previous MD simulations have also suggested that BODIPY-cholesterol can be considered a proper cholesterol analog as its membrane integration does not affect the membrane structure even though it exhibits significantly higher tilt angles than cholesterol^31^. This discrepancy might be due to the very low concentration of the BODIPY-cholesterol used in the simulation setup, which could allow for a buffering of the BODIPY-cholesterol effects in the otherwise unperturbed surroundings. Thus, these findings cannot be compared to our results on the CHIM effects on phospholipid order parameters that used much higher CHIM concentrations in the simulations. The high cholesterol similarity of CHIM as compared to BODIPY-cholesterol is also supported by an assessment of the tilt angle distribution of CHIM in relation to the cholesterol angle. Even in the pure DPPC/CHIM bilayers CHIM’s average tilt angle is only slightly increased and CHIM’s tilt becomes identical to that of cholesterol in the 1:4 CHIM/CHOL mixture (Supplementary Fig. 12). Together our results suggest that CHIM and CHIM-L are more closely related to natural cholesterol than BODIPY-cholesterol with respect to their membrane integration and cell labeling properties.

**Fig 5:**
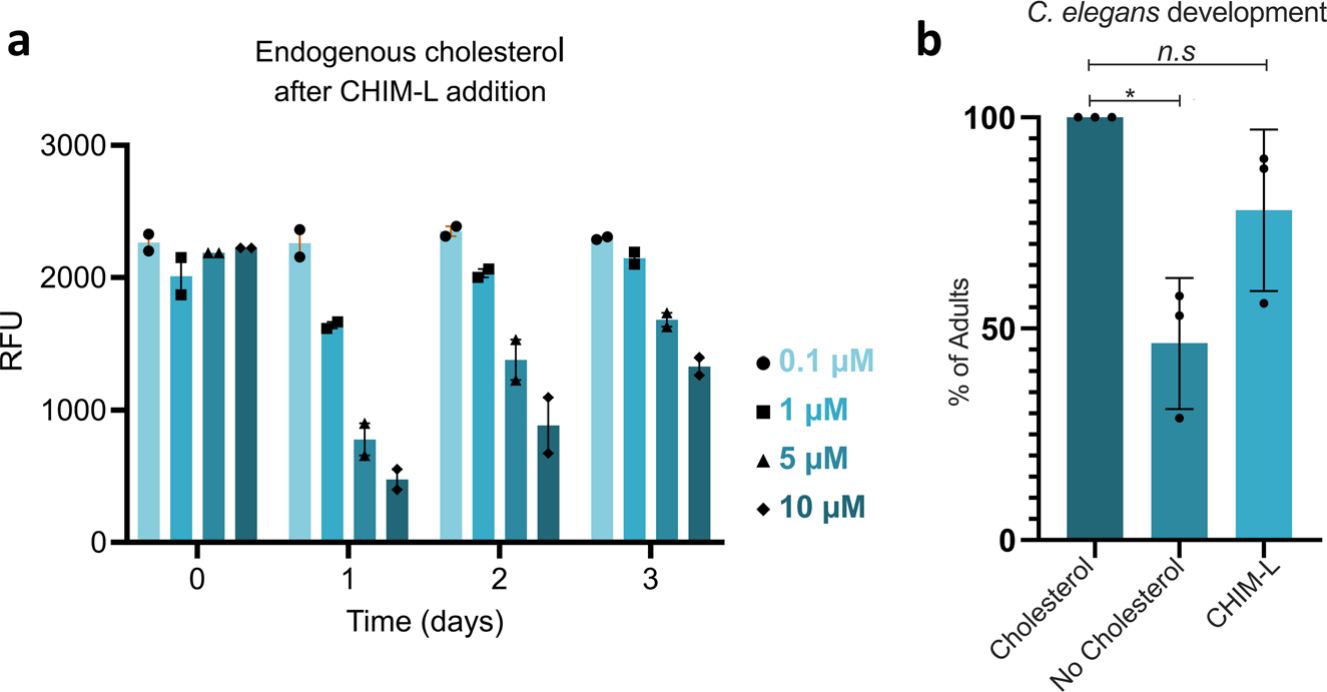
CHIM-L can functionally replace membrane cholesterol. **a** Relative levels of endogenous cholesterol in HUVEC treated with various concentrations of CHIM-L (0.1, 1, 5, and 10 µM) for 1.5 h at 37 °C. Following treatment, cells were washed three times in CHIM-free medium and analyzed directly or incubated up to 3 days in normal medium. Cholesterol concentration was quantified using a fluorometric assay (Amplex) and is given in relative fluorescence units (RFU). Error bars + SEM. **b** Effect of cholesterol removal and CHIM-L replacement diet on *C. elegans* development. Worms were investigated for two generations under standard cholesterol conditions (5 mg/l), no-added cholesterol, or CHIM-L (5 mg/l), and the number of worms that reached adulthood was quantified for the different feeding schemes (91 worms for cholesterol, 137 for no-added cholesterol, and 143 animals for CHIM-L). Error bars are SD. *p < 0.05.

Endogenous cellular cholesterol levels are kept under tight control, e.g., by regulation of the transcription of enzymes involved in cholesterol biosynthesis, and the cell balances internal and external cholesterol supply through enzymatic feedback regulation^32^. Therefore, we also assessed whether cells can sense CHIM levels as part of the cellular cholesterol balance and determined total cholesterol contents in cells loaded with different amounts of CHIM-L. CHIM-L was integrated into the PM of HUVEC at four different concentrations (0.1, 1, 5, and 10 µM) and the levels of endogenous cholesterol were measured after 1.5 h, as well as 1, 2, and 3 days, respectively, using an Amplex Red reaction assay^33,34^, that does not detect CHIM-L due to the absence of the 3-hydroxyl group. At 0.1 µM no significant effect on the level of endogenous cholesterol was visible, whereas higher CHIM-L concentrations (1, 5, and 10 µM) first caused a drop and then a restoration of total cholesterol levels (Fig. 5a). This suggests that cells sense the presence of CHIM-L in their membranes and adjust their total cholesterol levels accordingly. With time (2–3 days), CHIM-L is most likely metabolically degraded and/or externalized and endogenous cholesterol levels are restored. We also assessed whether this sensing of CHIM extends to the transcriptional control of cholesterol biosynthesis. Therefore, we analyzed the proteolytic cleavage of the sterol regulatory element-binding protein (SREBP) that liberates transcriptionally active SREBP fragments, which in turn activate transcription of target genes involved in cholesterol synthesis. Low cellular cholesterol levels that are sensed by the SREBP cleavage activating protein SCAP trigger cleavage and transcriptional activation whereas high cholesterol levels prevent cleavage by retaining the SCAP/SREBP complex in the ER^35^. HeLa cells were treated with CHIM-L at high concentrations (10 μM) and the SREBP signal in immunoblots was compared to that of cells either left untreated or treated with cholesterol or U18666A as a means to lower ER cholesterol levels. Supplementary Figure 13 shows that similar to cholesterol, CHIM-L appears to slightly reduce the proteolytic cleavage of SREBP.

### CHIM can functionally replace cholesterol in an animal model

The above data indicate that CHIM-L behaves similarly to cholesterol in mammalian cells. Therefore, we asked next whether CHIM-L could replace cholesterol in a whole animal. The nematode *Caenorhabditis elegans* represents an excellent model for studying different aspects of sterol function on the level of a whole organism, because, in contrast to mammals, *C. elegans* is a cholesterol auxotroph^36^. Cholesterol is essential for development and fertility and its depletion during *C. elegans* development induces loss of both motility and muscle mass together with a reduction of life expectancy^37^. Besides being a membrane component, cholesterol is involved in hormonal signaling during development and dauer formation^38–40^. In the laboratory, worms are grown in the presence of 5 mg/l of cholesterol^41^. Therefore, the feeding of CHIM-L instead of cholesterol can reveal whether CHIM-L can functionally replace natural sterols during development of the nematode.

To address this aspect, we grew worms for three generations in media containing either cholesterol, CHIM-L, or no-added sterols. Consistent with previous reports^42^, in the absence of dietary cholesterol, only 46% of the F1 generation worms reached adulthood and developed into fertile species, whereas this fraction increased to 78% in animals grown in the presence of CHIM-L (Fig. 5b). In the F2 generation, however, animals grown without cholesterol or in the presence of CHIM-L failed to develop into adults. The development of the F1 into adult worms demonstrates that CHIM-L can functionally replace cholesterol in membranes. We assume that the developmental arrest in the F2 generation is due to the inability of CHIM-L to function as a precursor for steroid hormones and thus inappropriate hormonal signaling. Nevertheless, our data indicate that CHIM-L can take over cholesterol functions in the membranes of a living animal.

## Discussion

Herein, we introduce imidazolium-based cholesterol analogs and report that they show cholesterol-like behavior in model and cellular membranes and can functionally replace cholesterol in the development of the F1 generation in *C. elegans*. Moreover, we conducted in-depth MD simulations to examine the influence of sterical differences between CHIM and CHOL as well as charge effects introduced by CHIM’s positive charge. We found that for sterol mixtures of 1:4 CHIM/CHOL, CHIM has no perturbing influence and even shows similar ordering and condensing characteristics as cholesterol. Similar ratios are likely to be met in cells loaded with CHIM or CHIM-L following the protocols used herein as the cells seem to be able to adjust their endogenous cholesterol content, which dropped following CHIM treatment, most likely to maintain the total cholesterol/CHIM homeostasis (Fig. 5a). We could also show that at physiological temperatures the membrane perturbing effects of CHIM can be largely attributed to its positive charge. They mainly take effect at concentrations above 15 mol% and are only observed in an otherwise uncharged lipid environment and hence are not relevant in cellular membranes. Importantly, the similarity to cholesterol is markedly increased when negatively charged lipids are present. This is corroborated by the GUV and GPMV experiments that showed no disturbing effect of CHIM-L when negatively charged phospholipids were included (GUV) or were naturally present (GPMV). Moreover, the GUV and GPMV experiments revealed that CHIM-L preferentially integrates into Lo membrane domains, thus exhibiting a phase separation behavior similar to that of natural cholesterol. This experimental finding is again in line with the computer simulations of a preformed DPPC/CHOL/CHIM domain in bilayers containing also DLiPC, where CHIM, in low concentrations, shows similar segregation behavior as cholesterol even without countercharged lipids. Together, the findings indicate that CHIM can integrate into biological membranes containing negatively charged lipids in a cholesterol-like manner, and it can be specifically labelled without perturbing its cholesterol-like membrane integration and partitioning.

Further improvement of our cholesterol probe is expected and will be driven by the MD simulations. For instance, the positive charge in CHIM could be removed chemically by generating a cholesterol analog bearing a polar, but uncharged imidazole headgroup instead of the permanently charged imidazolium salt. Alternatively, the design of an imidazolium salt with a more shielded permanent charge, for instance by choosing bulkier N-substituents, could represent an approach to reduce the undesired effect of charge on CHIM’s membrane integration. However, in this case, the supposedly beneficial partial shielding of the positive charge versus unfavorable, potentially disturbing interactions of the bulky headgroup with the membrane and the possible loss of CHIM’s cholesterol-resembling amphiphilic structure would need to be carefully balanced^10–13^.

Due to CHIM’s cholesterol-like nature it is readily integrated into cellular membranes in a manner most likely resembling natural cholesterol, and within cells, it follows the trafficking routes of the native counterpart. It is present in caveolae, which are known to contain high levels of cholesterol, and upon internalization is found in Rab5- and Rab7-positive compartments that also function as destinations for internalized natural cholesterol. The intracellular cholesterol distribution can be visualized by the cholesterol-binding sensor mCherry-D4H ectopically expressed following transfection. mCherry-D4H is present in the cytosol and therefore, it can only label cholesterol that resides in the cytosolic leaflet of the plasma membrane or of internal organelles. When expressed in CHIM-L treated cells mCherry-D4H shows a substantial but not full colocalization with the cholesterol analog. This supports the conclusion that CHIM-L resides in cholesterol-rich membranes/membrane domains but also suggests that not all cholesterol membrane pools are accessible to CHIM-L. Interestingly, upon overload, some CHIM-L most likely also accumulates in lipid droplets. As CHIMs cannot be esterified this suggests that the charged imidazolium ring does not preclude incorporation into this organelle. Future experiments, like isolating and analyzing CHIM-L-loaded lipid droplets, have to address this point.

All studies in model and cellular membrane complemented with the in-depth MD simulations predict that CHIM can function as a substitute for cholesterol in biological membranes and thus can be applied to study cholesterol trafficking and behavior in live cells following specific labeling at a site not affecting CHIM’s proper membrane integration. The functional equivalence as a membrane component is further corroborated by our finding that CHIM supports growth and development of an animal (*C. elegans*), which is cholesterol auxotroph and is reliant on exogenous addition of cholesterol in the diet.

In sum, we have shown that imidazolium-based cholesterol analogs (CHIMs) exhibit cholesterol-like behavior and can substitute natural cholesterol in native biological membranes and living cells. We, therefore, envision that this highly modifiable class of cholesterol analogs will serve as an important tool and flexible platform for the investigation of cholesterol-dependent membrane processes.

## Methods

### Synthesis of cholesterol analogs CHIM and CHIM-L

Cholesterol analogs CHIM and CHIM-L were synthesized as previously described^9^. The synthesis was performed starting from commercially available cholesterol that was converted into the corresponding 2,3-cholestandione in five steps. Subsequent formation of the respective imidazole, with either paraformaldehyde or 6-azidohexanal, and N-methylation yielded the herein investigated imidazolium-based cholesterol analogs CHIM and CHIM-L.

### Structure and parameters of the cholesterol analog

A relaxed structure of the cholesterol analog (CHIM) was obtained using Avogadro^43^ after an energy minimization with GaFF^44^. This structure was used as input for the CHARMM General Force Field (CGenFF) program version 1.0.0 to obtain parameters for the CGenFF version of 3.0.1^45^. Each parameter is provided with a penalty value to receive an estimate of its fitness. The atoms in CHIM were found to have an agreeable analog in already existing molecules in CGenFF, as visible through low penalty values. Only the nitrogen atoms and the attached methyl groups showed a slightly higher penalty, hence the fitness of these parameter values for this part of the molecule was further analyzed. As the best analogous molecule already parameterized in CGenFF is 7-methylguanosine, we compared those parameters to those of CHIM. We found that the overlap is sufficient for the bonded parameters. For the analysis of the fitness of partial charges, we received similar results following the common CGenFF procedure of parameterization. Therefore, and to avoid overparameterization, we decided to use the CGenFF program parameters^46,47^. To analyze charge effects, we additionally added a CHIM version with a zero net charge (CHIM-0, Supplementary Fig. 2) by rescaling the partial charges of all atoms within the imidazolium ring (excluding those charges of the alkyl hydrogens) by 1/8th of its partial charges. The parameters for CHIM-LX are based on the parameters of CHIM. The partial charges of the imidazolium hydrogen (H21) were incorporated into the partial charge of the alkyl carbon on its former H position. Alkyl carbons being more than a bond away from the cyanide group and all alkyl hydrogens were assigned the standard charges. All other charges and parameters were taken from the CGenFF output. A list of all used partial charges and angle and dihedral potential parameters with the respective penalty values is shown in Supplementary Tables 1, 2, and 3, respectively.

### System setup and analysis

All initial bilayers containing random mixtures of DPPC, DLiPC and cholesterol were prepared using the CHARMM-GUI web-based graphical interface^48^. To add CHIM to the configurations, cholesterol molecules were reconstructed to fit the relaxed CHIM template structure. The patched configurations were constructed from a mixture of DLiPC and CHOL, where CHOL again was replaced by CHIM and all center DLiPC molecules were reconstructed to DPPC to obtain a 2:3 ratio of DPPC/DLiPC. The resulting net charges of the systems were neutralized by exchanging random water molecules with either potassium or chloride anions in the system. In the experiments the counterion of CHIM was iodide due to its optimized synthesis pathway. In the simulations though, to avoid artifacts, we chose chloride as counterion as it is well established in the CHARMM force-field set and most commonly used. Since the aqueous biological environment most probably enables rapid dissociation and anion exchange, we don’t expect the choice of counterion to largely affect the properties of the CHIM analogs.

Calculation of the chain order parameter S employed α being the angle that is spanned by every second carbon atom in the alkyl chains and the average leaflet tilt. The average tilt angle is defined as the angle between the average of vectors from the first and last carbon atoms of phospholipid chains and the bilayer normal (*z*-axis of the simulation box).

To identify the position of CHIM or cholesterol the difference between the average z positions of the carbon atom attached to cholesterol’s hydroxyl group or the respective carbon atom in CHIM to the average z positions of the PL’s phosphor atoms in each bilayer leaflet was used.

All visualizations of the system configurations were created using VMD software^49^.

### Molecular dynamics simulations

All simulations were conducted using the CHARMM36 force field^50–52^ and Gromacs~2018 software package^53,54^. The systems were equilibrated using the 6-step established CHARMM-GUI parameter set, gradually decreasing restraints on lipid headgroup positions and chain dihedral angles (Supplementary Table 4). In the case of the pure DPPC bilayer, two additional 50 ns equilibration steps with position restraints of 1000 kJ/mol on the lipid head groups were performed to minimize leaflet intercalation. The first 50 ns were cutoff of each production run to avoid non-equilibrium statistics. A list of all systems with lipid composition and trajectory lengths is provided in Supplementary Tables 5, 6 and 7.

The TIP3P model was used as water model^55^. The Nosé-Hoover algorithm was used to maintain the temperature with a coupling constant of 1 ps, coupling bilayer and solvent separately^56^. To maintain the pressure at 1 bar the Parrinello-Rahman barostat was used with a semiisotropric coupling scheme, a coupling constant of 5 ps and compressibility of 4.5 × 10^−5^ bar-1^57^. Particle mesh Ewald electrostatics were used with a real-space cutoff of 1.2 nm^58^. The Lennard-Jones potential was shifted to zero between 1.0 and 1.2 nm, with a cutoff of 1.2 nm and the nonbonded interaction neighbor list was updated every 20 steps with a cutoff of 1.2 nm.

### Cell culture, microscopy, and transfections

HUVEC were purchased from Promocell, cultured in growth medium, a 1:1 mixture of ECGM2 and M199 + 100 i.u. heparin + 10% FCS (Sigma–Aldrich), supplemented with 0.015 μg/ml amphotericin B and 30 μg/ml gentamycin, and cultivated at 37 °C and 5% CO_2_. HeLa and MDCK type II cells were cultured in DMEM supplemented with 10% standardized fetal bovine serum (FBS Superior, Biochrom), 100 U/mL penicillin, and 0.1 mg/mL streptomycin.

For microscopy, HeLa, MDCK type II and HUVEC were seeded in eight-well-chambered glass bottom coverslips (ibidi). In the case of HUVEC, all dishes or slide surfaces were coated with collagen. Cell density was adjusted to reach ~70% confluency at the time of imaging. Live imaging was performed utilizing a LSM 780 confocal laser scanning microscope (CLSM, Carl Zeiss) equipped with an Argon-Ion laser (LASOS) and a Plan-Apochromat ×63 /1.4 oil immersion differential interference contrast objective lens (Carl Zeiss).

HeLa and MDCK cells were transfected with Lipofectamine™ 2000 (Thermo Fischer Scientific) following the manufacturer’s guidelines, HUVEC were electroporated for plasmid DNA transfection (1–10 μg per 20 cm^2^ nearly confluent cells) as described^59^. Cells were treated with 5 µM CHIM-L-fluorophore (1:1) 24 h post transfection for 30 min at 37 °C in full growth medium. The plasmids used were described before: Lamp1-mGFP^60^, mCherry-D4H^28^, Cav1-mCherry^61^, Cav1-mApple^62^, EGFP-Rab5^63,64^, Rab7a-EGFP^65^. All cell lines were regularly tested for mycoplasma contamination.

### Cell surface click-reaction and uptake of preclicked CHIM-L

Bioorthogonal click reactions to label CHIM-L incorporated into the plasma membrane of HeLa cells, MDCK or HUVEC were performed in a copper free manner with Click-iT-DIBO Alexa Fluor® 647, click-iT DIBO Alexa Fluor® 488 (Thermo Fischer Scientific), or AFDye 405 DBCO (Click Chemistry Tools). Therefore, cells were incubated for 20 min with 5 µM of CHIM-L (prepared in full medium, DMEM for HeLa and MDCK or mixed ECGM2 and M199 medium for HUVEC) either at room temperature or on ice (in the latter case the medium also contained 20 mM HEPES, pH 7.4) and then washed 4 times at the respective temperature. Labeling via click reaction was then performed by adding 5 μM of the respective Click-iT DIBO-dye in the same medium for 20 min on ice, followed by washing the cells four times with PBS+/+ (containing Ca^2+^ and Mg^2+^) and two times with the respective growth medium containing 20 mM HEPES. In this application procedure, CHIM-L most likely associates with serum proteins before it is transferred to the cell membrane.

For incorporation of dye-labeled CHIM-L, HeLa, MDCK, or HUVEC were cooled down on ice for 20 min and then treated for an additional 20 min on ice with 5 μM of a preclicked 1:1 mixture of CHIM-L and the alkyne-fluorophore, followed by four times washing with PBS+/+ and two times with the respective growth medium containing 20 mM HEPES, pH 7.4.

For BODIPY-cholesterol and/or CHIM-L (AF405) labeling HeLa cells or HUVEC were cooled to room temperature for 20 min in full growth medium containing 20 mM HEPES, pH 7.4. AF405-labeled CHIM-L and BODIPY-cholesterol were diluted to 5 µM in full growth medium containing 20 mM HEPES, pH 7.4, and then given to the cells, which were then subjected to live cell imaging.

HUVEC cells were exposed to the NPC1 inhibitor U18666A (Sigma–Aldrich) at 2 µg/mL overnight, and lysosomes were labeled by adding 75 nM of LysoTracker™ Red DND-99 (Molecular Probes) in full growth medium, which was given for 30 min prior imaging. CHIM-L and BODIPY-cholesterol incubation was carried out as described above.

### Lipid droplet labeling

Lipid droplets were labeled with Bodipy™ 493/503 (Thermo Fischer Scientific)^66^ by incubating cells with a final dye concentration of 5 µM diluted in the appropriate growth medium for 1 h at 37 °C, followed by three times washing with medium. Before or after treatment, the cells were given 2.5 µM CHIM-L preclicked with DIBO-Alexa Fluor.

### GUVs

The lipids 1-palmitoyl-2-oleoyl-sn-glycero-3-phospho-L-serine (sodium salt) (POPS), 1,2-dilinoleoyl-sn-glycero-3-phosphocholine (DLiPC), 1,2-dipalmitoyl-sn-glycero-3-phospho-choline (DPPC), 1-palmitoyl-2-oleoyl-glycero-3-phosphocholine (POPC) were purchased from Avanti Polar Lipids.

Giant unilamellar vesicles (GUVs) were generated employing the gentle agarose swelling method^20,67^. Briefly, lipid mixtures containing POPS:DLiPC:DPPC:CHIM-L (14.29:42.85:28.57:14.29) were prepared from 5 mM stock solutions and added to a thin film of agarose, which was prepared in a two-well ibidi slide by addition of a 1% w/v solution of ultra-low gelling agarose in double distilled H_2_O at 60 °C. The mixture was allowed to dry for 30 min in vacuum and was then equilibrated in HEPES buffer for 1 h, followed by clicking with AF488 (5 µM) and labeling with FAST DiI (5 µM).

### Quartz crystal microbalance with dissipation (QCM-D)

QCM-D was performed as described before^68^ employing small unilamellar vesicles (SUVs) composed of POPC:POPS:CHIM (60:20:20) to form the solid-supported bilayer. AnxA2 binding was accomplished in presence of 250 µM Ca^2+^, in HBS buffer (10 mM HEPES, 150 mM NaCl, pH 7.8 at 25 °C). Experiments were carried out at a constant temperature of 20 °C.

### Cholesterol quantification via Amplex® Red assay

Membrane cholesterol levels were determined in cultured HUVEC after treatment with different CHIM-L concentrations (ranging from 0.1 to 10 µM). Quantification employed the commercial Amplex Red Cholesterol Kit (Invitrogen) according to the manufacturer’s instructions. Briefly, samples were diluted in reaction buffer and mixed with an equivalent volume of the Amplex Red working solution (300 µM Amplex Red, 2 U/ml horseradish peroxidase, 2 U/ml cholesterol oxidase and 0.2 U/ml cholesterol esterase). This resulted in a final concentration of 150 µM Amplex Red reagent in the reaction mix and a final volume of 100 µl per well. Samples were incubated for 90 min at 37 °C, subsequently washed three times, and fluorescence was determined by using a fluorescent microplate reader. All plates were protected from light and the data (*n* = 2) were plotted with Graphpad Prism 8.

### GPMV isolation and labeling

GPMVs were isolated from cultured HUVEC as previously described^18,69^. Briefly, cells were cultured up to 70% confluency, washed twice with GPMV buffer (150 mM, NaCl, 2 mM, 10 mM HEPES, CaCl2, pH 7.4) and incubated with GPMV vesiculant chemical mix (GPMV buffer plus 25 mM PFA and 2 mM DTT) for 1 h at 37 °C. Subsequently, cells were placed at 4 °C and treated with 500 µM deoxycholic acid (Sigma–Aldrich) for 30 min to cause phase separation. GPMVs were then collected from the supernatant and transferred into a plastic vessel containing CHIM-L preclicked to Alexa Fluor 488 and FAST DiI, all used at a final concentration of 5 µM. Next day, GPMVs present at the bottom of the vessel were imaged at room temperature after transferring the vesicles into poly-l-lysine 0.1 % (w/v) (Sigma–Aldrich) coated eight-well μ-slide glass bottom dishes (Ibidi). The respective GMPV isolations were performed in three independent experiments.

### SREBP1 detection by western blot

HeLa cells were grown in 10 cm dishes to full confluency and incubated with either CHIM-L or cholesterol (Avanti Polar Lipids), both at 10 µM in full growth medium for 90 min, or with U18666A (Sigma–Aldrich) at 2 µg/mL overnight in full growth medium. Cells were scraped in cold lysis buffer (50 mM Tris, pH 7.4, 150 mM NaCl, 1 mM EDTA, 1% NP-40, 0.5% sodium deoxycholate, and 1× Complete EDTA-free Proteinase Inhibitor Cocktail) on ice and the cell suspension was sonicated for 1 min (amplitude 100, 0.5 cycle). After centrifugation (15,000 × *g*, 1 min, 4 °C), the protein concentration of the cell lysates was measured using BCA protein assay reagent (Thermo Fisher Scientific). Equal amounts of total protein (30 µg) in Laemmli sample buffer were subjected to 12% SDS–PAGE and transferred to nitrocellulose membranes (GE Healthcare Life Science). Membranes were blocked with 5% nonfat milk in TBS containing 0.1% Tween-20 (TBST) (1 h, RT) and incubated with mouse monoclonal anti-SREBP1 antibodies (Abcam Biochemicals, ab3259) overnight at 4 °C. After washing and treatment with goat anti-mouse IR dye–conjugated secondary antibodies (1 h, RT), immunoreactive bands were visualized using the Odyssey Infrared Imaging System (Li-COR Bioscience) and quantified using Image Studio Lite (Li-COR Bioscience).

### Animal experimentation

*C. elegans* N2 worms were cultured and maintained at 20 °C as described previously^41^. The agar of the NGM plates was replaced with agarose, and cholesterol was either replaced with ethanol or CHIM-L. For developmental and survival assays, eggs from 1-day adult worms were hatched directly on plates containing OP50 bacteria and cholesterol, ethanol, or CHIM-L, respectively. One day after worms reached adulthood adult worms were removed and L1 worms were transferred to fresh plates with either cholesterol, CHIM-L, or ethanol. The developmental stage was assessed after 72 h; worms with eggs were considered adults.

### Statistics and reproducibility

In the *C. elegans* experiments, at least 90 worms from three independent experiments were analyzed, an ANOVA test was performed with Tukey’s test using Graphpad Prism 8. Errors of the mean neighborhood (Fig. 3b) were determined using the blocking method.

### Reporting summary

Further information on research design is available in the Nature Research Reporting Summary linked to this article.

## Supplementary information

Supplemental Information

Description of Supplementary Files

Supplementary Data 1

Reporting Summary

## Data Availability

The data that support the findings of this study are available on request from the corresponding authors V.G., F.G., and A.H. Source data is available as Supplementary Data 1.
