# Author Correction: CHIMs are versatile cholesterol analogs mimicking and visualizing cholesterol behavior in lipid bilayers and cells

**DOI:** 10.1038/s42003-021-02570-8

**Published:** 2021-08-26

**Authors:** Anna L. L. Matos, Fabian Keller, Tristan Wegner, Carla Elizabeth Cadena del Castillo, David Grill, Sergej Kudruk, Anne Spang, Frank Glorius, Andreas Heuer, Volker Gerke

**Affiliations:** 1 Institute of Medical Biochemistry, Center for Molecular Biology of Inflammation, University of Münster, Münster, Germany; 2 Physical Chemistry Institute, University of Münster, Münster, Germany; 3 Center for Multiscale Theory and Computation (CMTC), Münster, Germany; 4 Institute of Organic Chemistry, University of Münster, Münster, Germany; 5 Biozentrum, University of Basel, Basel, Switzerland

**Keywords:** Computational chemistry, Cell biology

Correction to: *Communications Biology* 10.1038/s42003-021-02252-5, published online 11 June 2021.

In the original version of the Supplementary information associated with this Article, there was an error in Supplementary Figure 13 where a black background colour obscured the graph in the figure. This has now been corrected in the PDF and HTML versions of the Article.

