# Supplementary material for: CHIMs are versatile cholesterol analogs mimicking and visualizing cholesterol behavior in lipid bilayers and cells
Source: Commun Biol. 2021 Jun 11;4:720. doi: 10.1038/s42003-021-02252-5 (PMC8196198; doi:10.1038/s42003-021-02252-5)
Supplement: Supplementary file 1 — Supplemental Information [file 42003_2021_2252_MOESM1_ESM.pdf]

Supplementary Information for

CHIMs are versatile cholesterol analogs mimicking and visualizing cholesterol behavior in lipid bilayers and cells

Anna L. L. Matos [1 §], Fabian Keller [2, 3 §], Tristan Wegner [4 §], Carla Elizabeth Cadena del Castillo [5], David Grill [1], Sergej Kudruk [1], Anne Spang [5], Frank Glorius [4*], Andreas Heuer [2, 3*], Volker Gerke [1*]

[1] Institute of Medical Biochemistry, Center for Molecular Biology of Inflammation, University of Münster, Von-Esmarch-Strasse 56, D-48149 Münster, Germany.
[2] Physical Chemistry Institute, University of Münster, Corrensstrasse 28/30, D-48149 Münster, Germany.
[3] Center for Multiscale Theory and Computation (CMTC), Corrensstrasse. 40, D-48149 Münster, Germany.
[4] Institute of Organic Chemistry, University of Münster, Corrensstrasse 40, D-48149 Münster, Germany.
[5] Biozentrum, University of Basel, Klingelbergstrasse 70, CH-4056 Basel, Switzerland.

[§] These authors contributed equally
* Correspondence: Volker Gerke, Andreas Heuer, Frank Glorius
E-mail:
gerke@uni-muenster.de
andheuer@uni-muenster.de
glorius@uni-muenster.de

Supplementary Note

Applicability of simulations of CHIM in comparison to CHIM-L

Due to their non-trivial implementation of dihedral potentials, the inclusion of linear moieties to the CHARMM force-field containing at least four atoms like azide groups is rather challenging. Therefore, to date only a few groups have derived parameters for such moieties for the CHARMM force-field[1]. In the simulations, we characterized the difference between cholesterol and CHIM induced by switching the cholesterol hydroxyl group to an imidazolium salt residue, without taking into account the linker present in CHIM-L because of the above-mentioned problematic inclusion of azide groups. Therefore, to obtain an estimate of the impact of a linker to the investigated CHIM characteristics we analyzed a slightly modified CHIM-L (CHIM-LX, Supplementary Figure 1A) where cyanide was chosen as an analog of the azide group. We found that CHIM-LX has a slightly higher perturbing effect on DPPC order parameters (Supplementary Figure 1B), which is also reflected in a slightly decreased bilayer thickness when comparing the density profiles for CHIM and CHIM-LX bilayers (Supplementary Figure 1D). CHIM-LX is slightly more tilted than CHIM even though in the mixtures of ANALOG (CHIM or CHIM-LX) with cholesterol this difference is barely visible (Supplementary Figure 1C). Thus, even though CHIM-LX exhibits a slightly higher perturbance on the bilayer structure, the differences in membrane characteristics between CHIM-LX and CHIM are generally very small, and the conclusions obtained in this work apply to both CHIM and CHIM-LX.

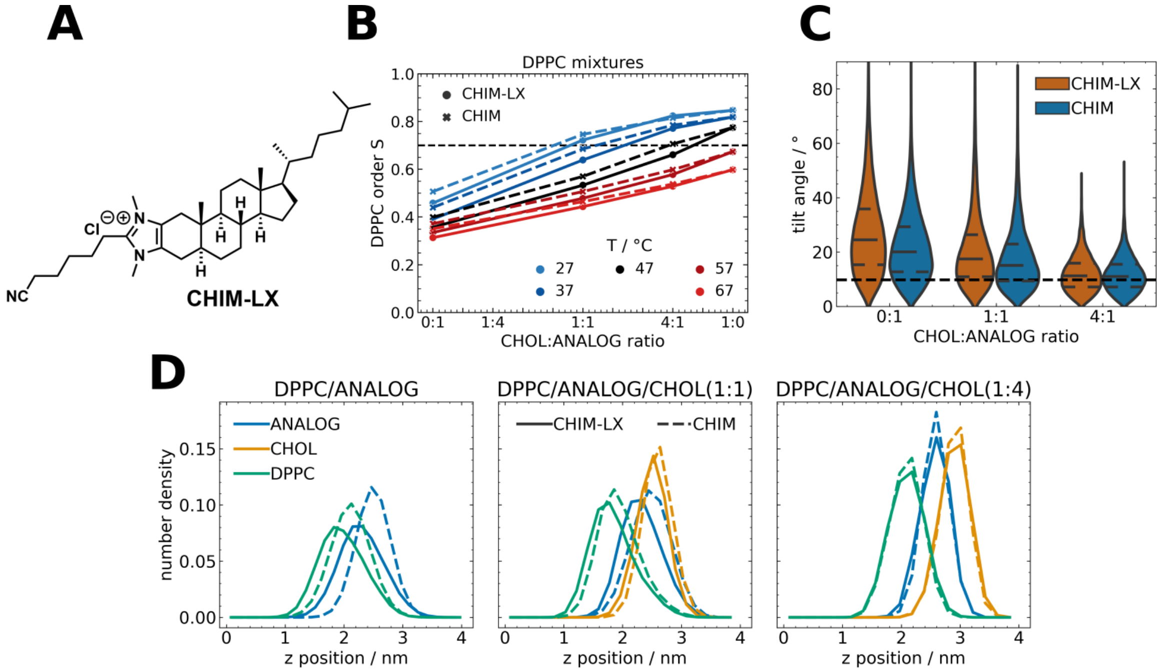

Supplementary Figure 1. Comparison membrane properties of CHIM-LX containing an alkyl linker with a cyanide group (A) and CHIM. (B) shows the average DPPC order parameter as a function of CHOL-ANALOG (CHIM or CHIM-LX) ratio, (c) sterol tilt angle distribution, with the dashed line indicating the average tilt angle of CHOL in a pure DPPC/CHOL bilayer and (D) density profiles of the z-position of DPPC (P atom as reference) and CHIM/ANALOG (C3 carbon atom as reference) for one leaflet.

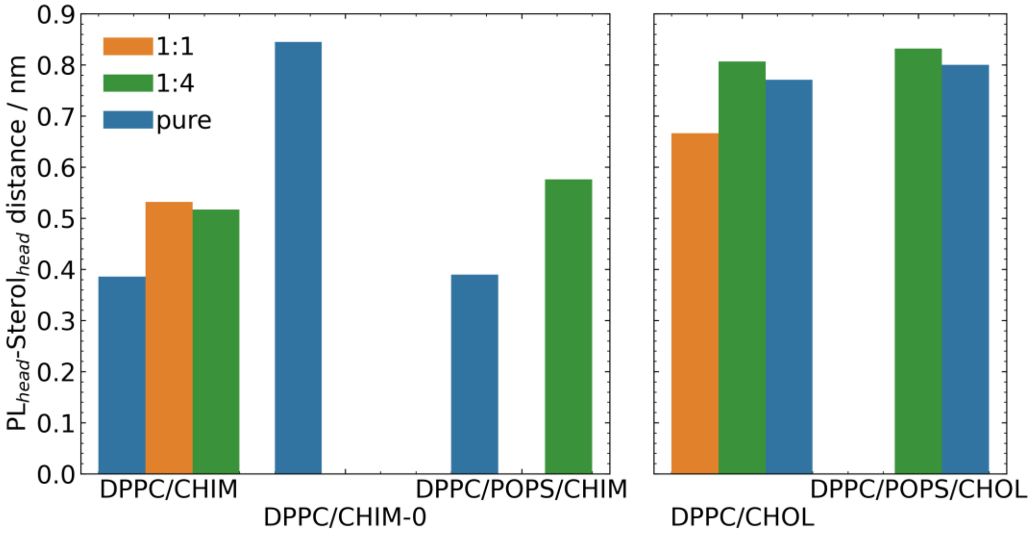

Supplementary Figure 2. Average distances of DPPC's phosphate atom to the carbon atom at position 3 of cholesterol (right) or the respective carbon position of CHIM (left) within simulations of different bilayer compositions at 27 °C. The color code indicates the CHIM:CHOL ratio within the bilayers, while pure indicates either a bilayer composition of DPPC with only CHIM or only CHOL.

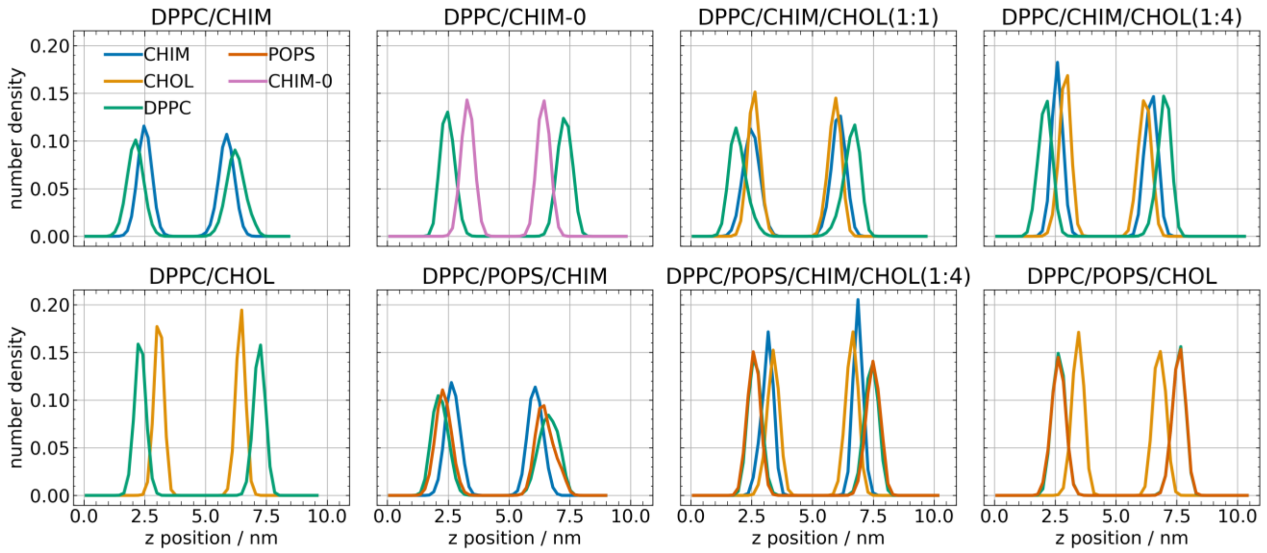

Supplementary Figure 3. Density profiles of the z-position of PLs (P atom as reference) and CHOL/CHIM/CHIM-0 (C3 carbon position of steroid ring numbering) for different bilayer compositions at simulation temperatures of 27 °C.

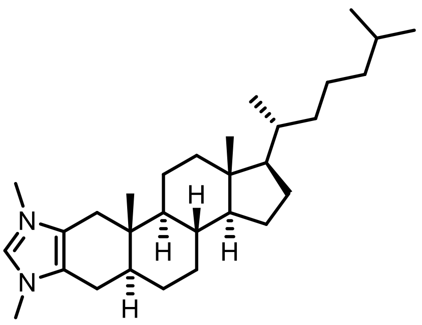

CHIM-0

Supplementary Figure 4. Structure of the fictive CHIM derivative CHIM-0.

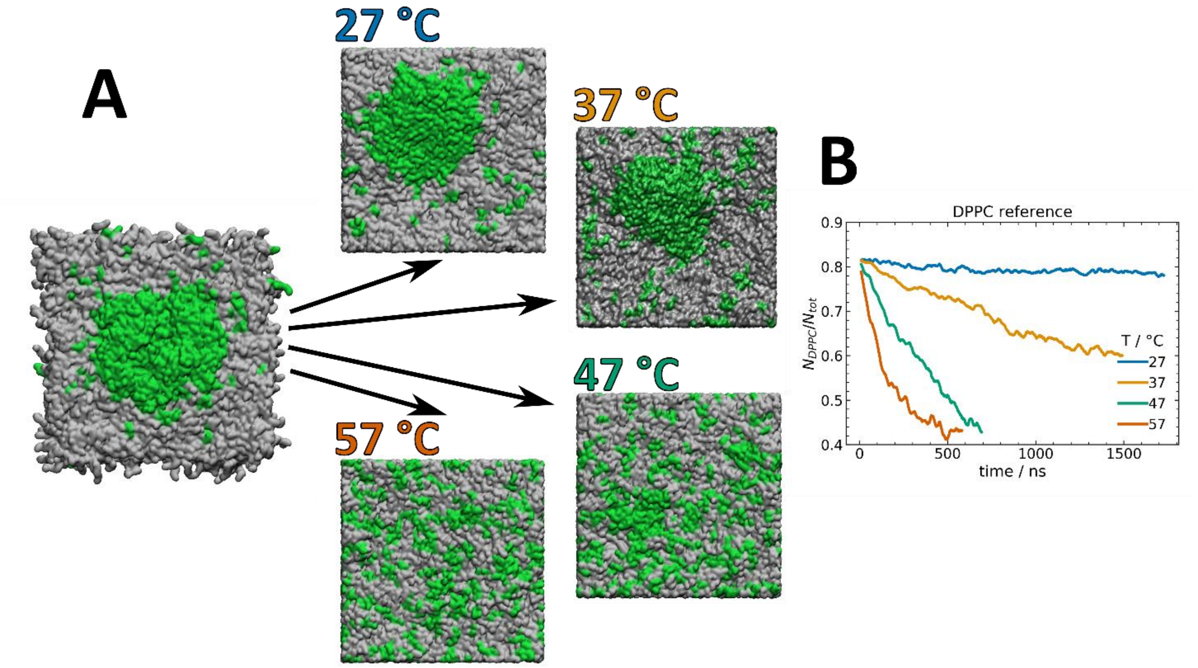

Supplementary Figure 5. A. Top view on a DPPC/DLiPC (4:6) bilayer (left). DPPC molecules are shown in green and DLiPC in grey. The left snapshot shows the initial configuration, and the right snapshots show the final configurations after a simulation at the respective temperature. **B.** Time series displaying the number of DPPC neighbors over the total number of PL neighbors around DPPC molecules for the corresponding simulations.

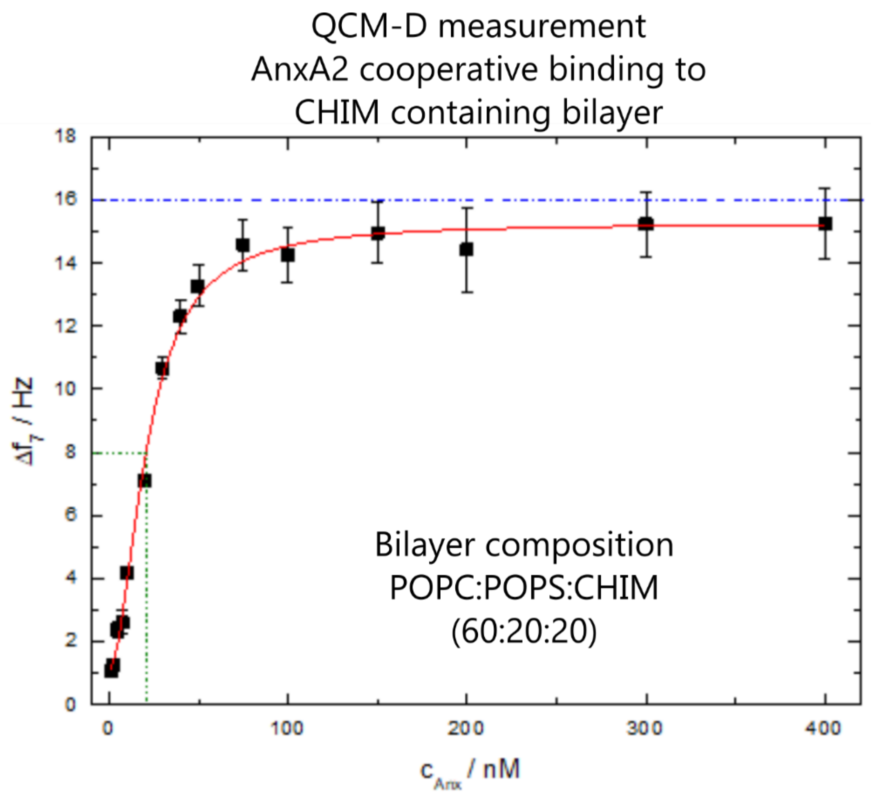

Supplementary Figure 6. QCM-D recordings of the AnxA2-lipid interaction. Saturation isotherm of the binding of AnxA2 to a POPC:POPS:CHIM (60:20:20) bilayer in the presence of 250 μM Ca^{2+}. The curve is the nonlinear regression (Hill expansion of the Langmuir binding isotherm)[2]. The upper blue line indicates the saturation value of adsorption.

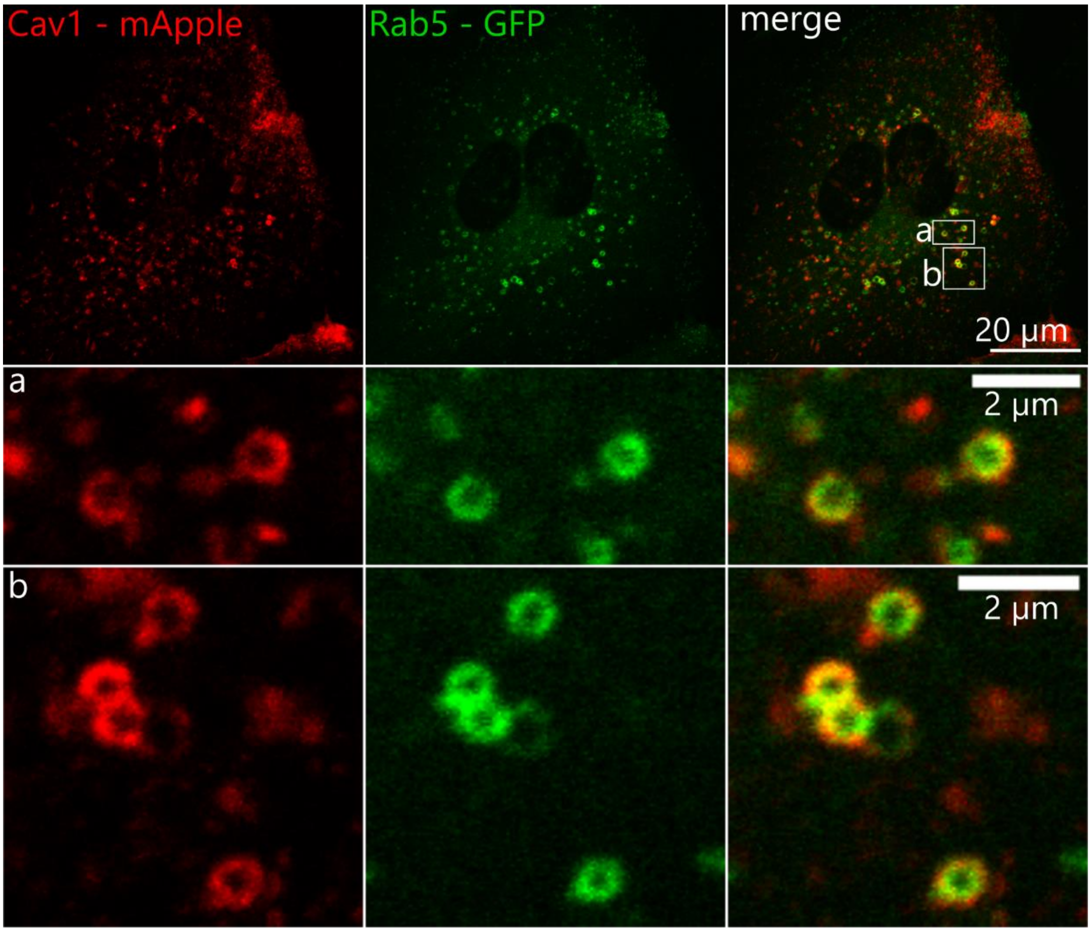

Supplementary Figure 7. Stills of live cell microscopy recordings of HUVEC ectopically expressing Caveolin 1 (Cav1)-mApple and Rab5-GFP. Areas **a** and **b** are shown at higher magnification in the bottom rows. Note that some Cav1 is present in internal structures identified as early endosomes by Rab5-GFP labeling.

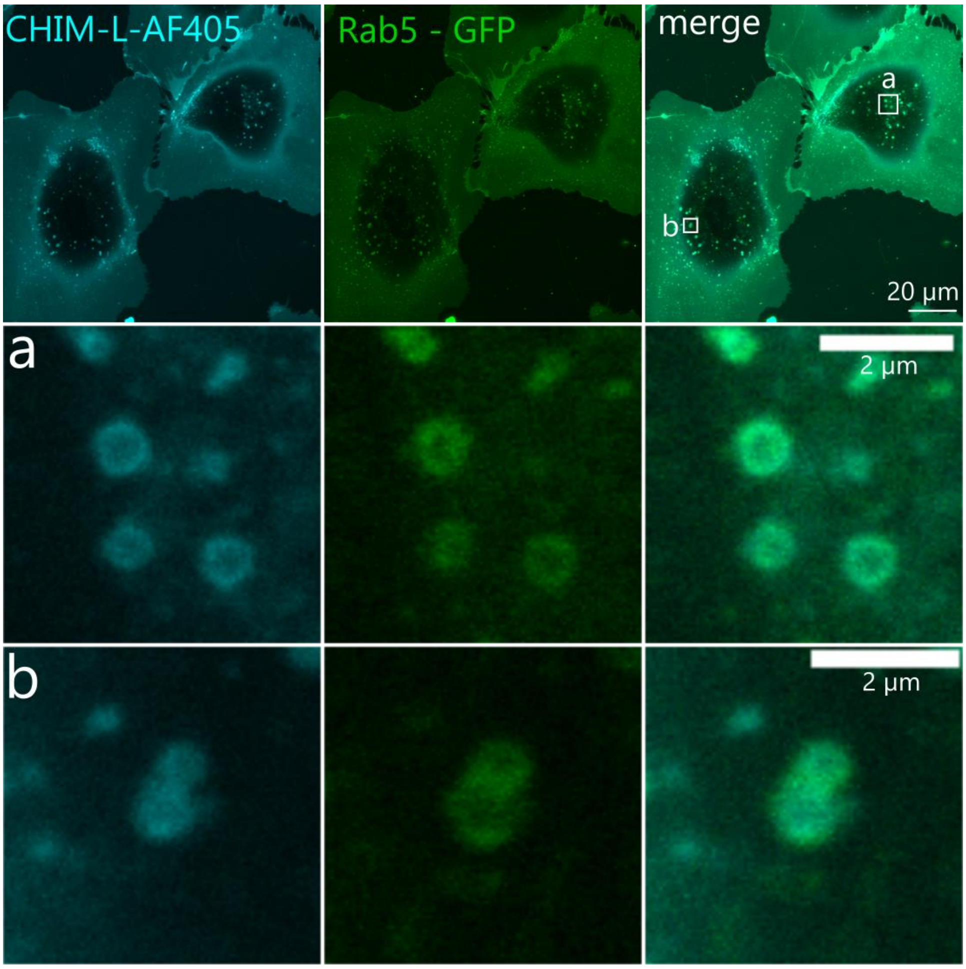

Supplementary Figure 8. Stills of live cell microscopy recordings of HUVEC ectopically expressing Rab5-GFP. Cells were incubated with CHIM-L pre-clicked to AF405 for 20 min prior to imaging. Areas **a** and **b** depicting a region above the nucleus (darker label) are shown at higher magnification in the bottom rows.

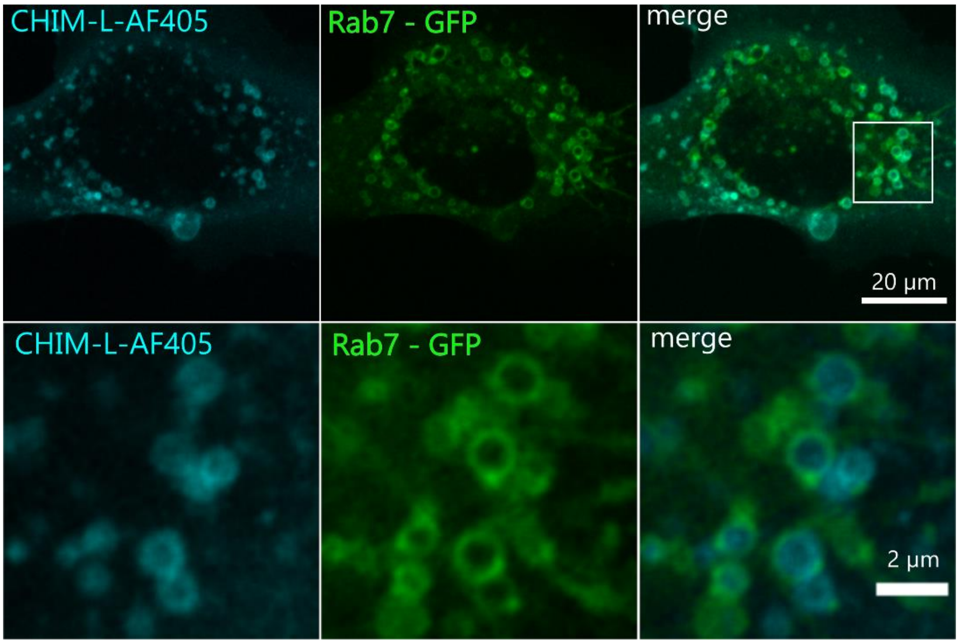

Supplementary Figure 9. Stills of live cell microscopy recordings of HUVEC ectopically expressing Rab7-GFP. Cells were incubated with CHIM-L pre-clicked to AF405 for 20 min prior to imaging. The inset is shown at higher magnification at the bottom.

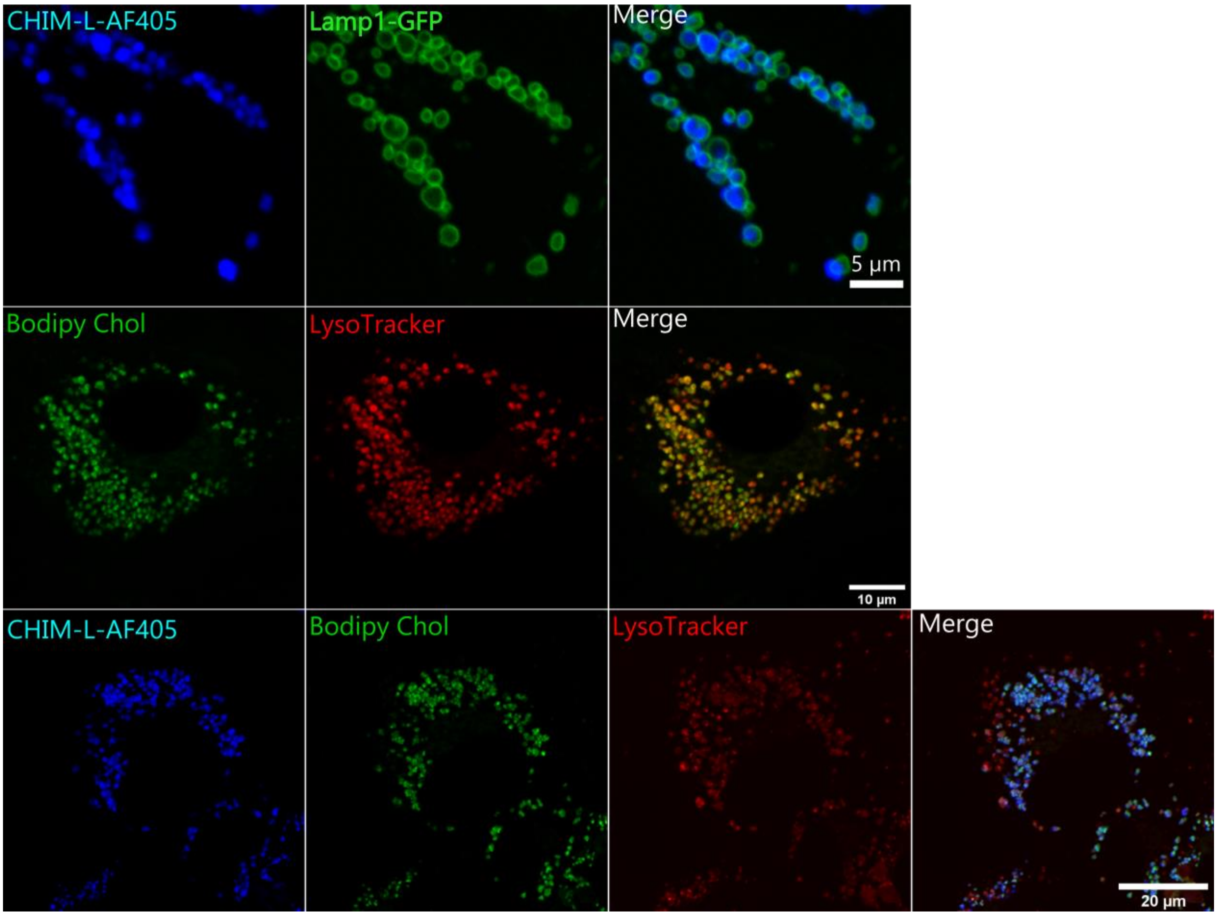

Supplementary Figure 10. Stills of live cell microscopy of HUVEC. Upper row, cells ectopically expressing Lamp1-EGFP as late endosome marker were treated overnight with the NPC1 inhibitor U18666A and incubated with 5 µM CHIM-L pre-clicked to AF405 for 20 min prior to imaging. Middle and bottom row, cells were incubated either with 5 µM Bodipy-cholesterol alone (middle row) or together with 5 µM CHIM-L pre-clicked to AF405 (bottom row) in full medium for 60 min, treated overnight with U18666A and, prior to imaging, incubated with 75 nM Lysotracker™ Red DND-99 for 30 min.

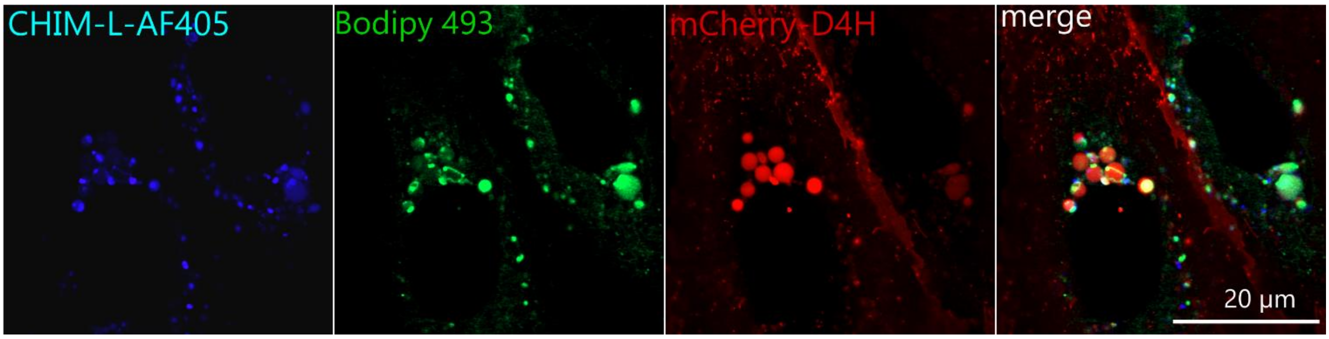

Supplementary Figure 11. Live cell microscopy showing lipid droplets in primary human endothelial cells (HUVEC). Cells were transfected with a mCherry-D4H construct and incubated for 20 h with AF405 labeled CHIM-L. Lipid droplets were stained with Bodipy 493.

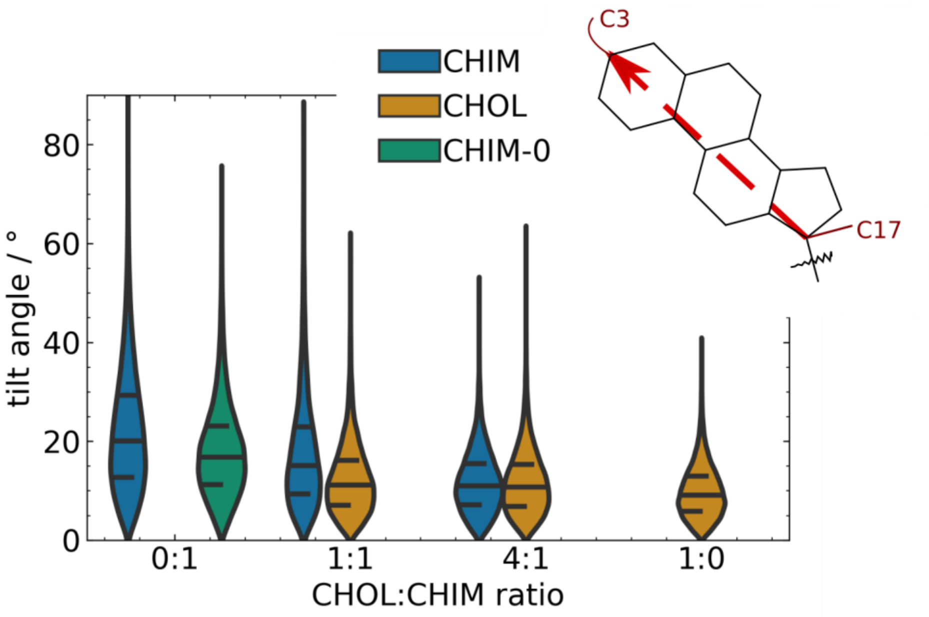

Supplementary Figure 12. Tilt angle distributions of CHOL, CHIM or CHIM-0 in different bilayer compositions with DPPC. The sketch in the upper right illustrates the reference vector in each sterol body to the bilayer normal (z-axis) spanning positions C17 to C3 of cholesterol steroid body.

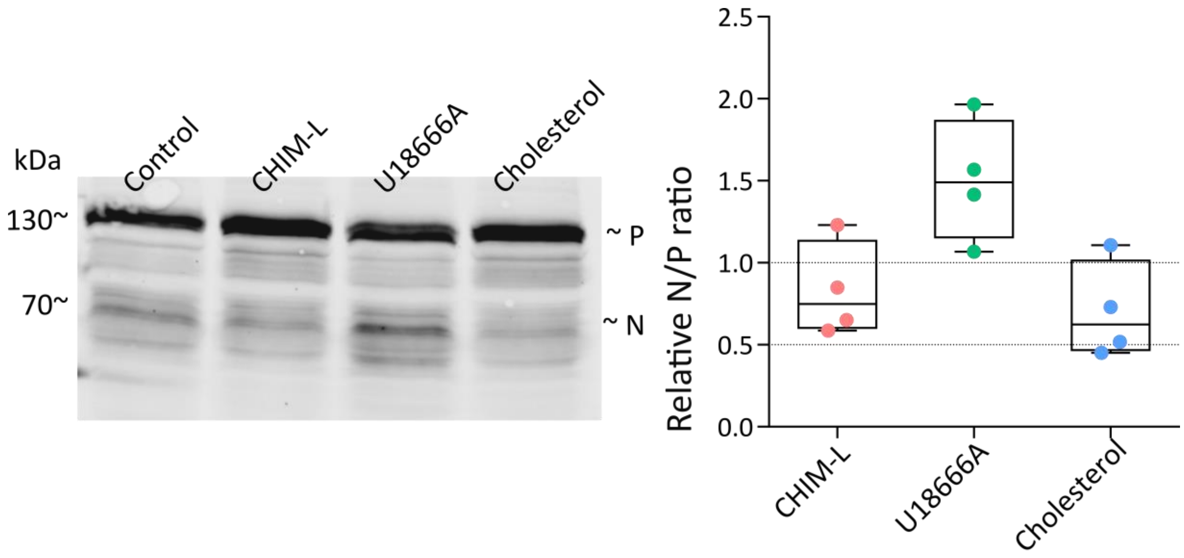

Supplementary Figure 13. SREBP1 Western blot and quantification. HeLa cells were left untreated (Control) or treated with CHIM-L (10 µM for 90 min), U18666A (2 µg/ml overnight), or cholesterol (10 µM for 90 min) and the respective cell lysates were subjected to Western blotting with anti-SREBP antibodies. One examplary blot is shown on the left side with the migration positions of the SREBP1 precursor (~P, at ~130 kDa) and the proteolytic fragment of SREBP1 that translocates into the nucleus (~N, at ~70 kDa) marked. Results of four independent experiments were quantified and plotted in the graph shown on the right side as relative N/P ratio in relation to the control (dashed line set at 1). Note that treatment with CHIM-L or cholesterol reduces the amount of cleaved SREBP1, whereas U18666A treatment that traps cholesterol in late endosomes results in an increased SREBP proteolysis.

Supplementary Table 1. Partial charges and atom types taken from CGenFF program for CHIM and the respective penalty values and the rescaled partial charges of CHIM-0. On the right the atom numbering of the structure of CHIM/CHIM-0 for reference are shown.

atom	atomtype	charge CHIM/CHIM-LX	charge CHIM-0	penalty
C1	CG2R51	0.192	0.1	20.142
C2	CG2R51	0.192	0.1	20.262
C3	CG321	-0.14	-0.18	standard
C4	CG321	-0.14	-0.18	standard
C17	CG311	-0.09	-0.09	21.033
C18	CG301	0.0	0.0	5.717
C19	CG331	-0.27	-0.27	8.665
N1	NG2R52	-0.511	-0.605	adapted
N2	NG2R52	-0.511	-0.605	adapted
C20	CG2R53	0.319	0.23	23.694
C21	CG334	0.17	0.078	adapted
C22	CG334	0.17	0.078	adapted
H33	HGR53	0.179	0.087	20.512
C31	CG321	-0.01	—	adapted
C32	CG321	-0.18	—	standard
C33	CG321	-0.18	—	standard
C34	CG321	-0.18	—	standard
C35	CG321	-0.18	—	standard
C36	CG1N1	0.36	—	adapted
N3	NG1T1	-0.46	—	0.0

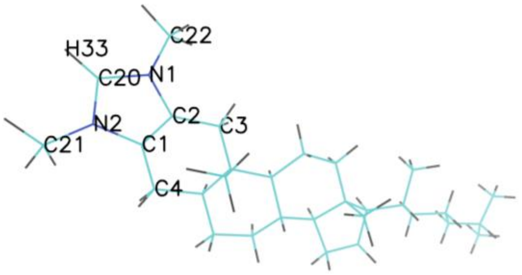

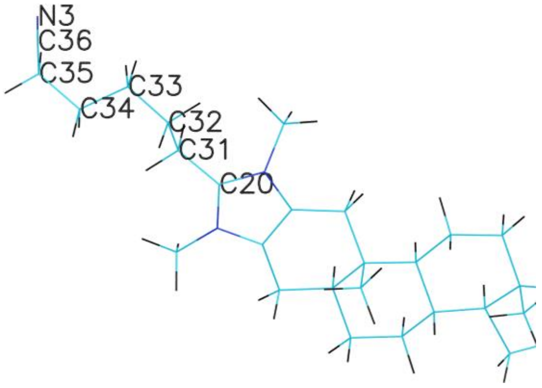

Supplementary Table 2. Bonded angle parameters for the angle potential of $V_{ang}(\Theta) = K_{\theta}(\Theta - \Theta_0)^2$ and $V_{UB}(S) = K_{UB}(S - S_0)^2$ used for CHIM and CHIM-0 from the CGenFF program output along with the respective penalty values.

atoms	K_α / kcal/mol/rad	α_0 / °	K_{UB} / kcal/mol/Å^2	S_0	penalty
CG2R51 NG2R52 CG334	25.0	124.9	15.0	2.13	141
CG2R53 NG2R52 CG334	25.0	127.1	15.0	2.09	141

Supplementary Table 3. Dihedral angle parameters for the dihedral potential $V_{dih}(S) = K\chi\left(1 + \cos(\mathrm{n}(\chi - \delta))\right)$ used for CHIM and CHIM-0 from the CGenFF program output along with the respective penalty values.

atoms	Kχ / kcal/mol	n	δ / °	penalty
CG2R51 CG2R51 CG321 CG301	0.2000	1	0.0	1.2
CG2R51 CG2R51 CG321 CG301	0.2700	2	0.0	1.2
CG2R51 CG2R51 CG321 CG301	0.0000	3	0.0	1.2
NG2R52 CG2R51 CG321 CG301	0.1900	3	0.0	1.2
CG2R51 CG2R51 NG2R52 CG334	60.000	2	180.0	32.4
CG321 CG2R51 NG2R52 CG334	25.000	2	180.0	114.5
CG311 CG301 CG321 CG2R51	0.0400	3	0.0	18.0
CG331 CG301 CG321 CG2R51	0.0400	3	0.0	16.5
CG301 CG311 CG321 CG2R51	0.0400	3	0.0	11.2
CG321 CG311 CG321 CG2R51	0.0400	3	0.0	9.3
HGA3 CG334 NG2R52 CG2R51	0.1500	3	180.0	82.0
HGA3 CG334 NG2R52 CG2R53	0.1500	3	180.0	82.0

Supplementary Table 4. Ensembles and force constants used during the equilibration steps on head group position and chain dihedrals.

step	time / ps	ensemble	restraints head group z position (kJ/mol)	restraints chain dihedral angle (kJ/mol)
1	25	NVT	1000	1000
2	25	NVT	1000	400
3	25	NVT	400	200
4	100	NpT	200	200
5	100	NpT	40	100
6	100	NpT	0	0

Supplementary Table 5. Compositions, trajectory lengths and temperatures of simulations of mixtures with the phospholipids DPPC or DPPC and POPS and one of or a mixture of CHIM and CHOL.

phospholipid	compound	sterol content (mol%)	temperatures (°C, 10 °C steps)	length (ns)
DPPC	CHOL	30	27-67	>500
DPPC	CHIM	30	27-67	600
DPPC	CHIM/CHOL (1:1)	30	27-67	600
DPPC	CHIM/CHOL (1:4)	30	27-67	600
DPPC	CHIM-0	30	27-67	600
DPPC	--	0	27-47	2000
DPPC	--	0	57-67	>1000
DPPC:POPS (2:1)	--	0	27-67	600
DPPC:POPS (2:1)	CHOL	20	27-67	600
DPPC:POPS (2:1)	CHIM	20	27-67	600
DPPC:POPS (2:1)	CHIM-0	20	27-67	600
DPPC:POPS (2:1)	CHOL:CHIM (1:4)	20	27-67	600

Supplementary Table 6. Compositions, trajectory lengths and temperatures of simulations of DPPC patches in DLiPC with and without sterol molecules.

phospholipid (6:4)	compound	ratio	sterol content (mol%)	T (°C)	length (µs)
DLiPC:DPPC	--	6:4	0	27	1.7
DLiPC:DPPC	--	6:4	0	37	1.5
DLiPC:DPPC	--	6:4	0	47	0.7
DLiPC:DPPC	--	6:4	0	57	0.5
DLiPC:DPPC	CHIM	6 : 4 : 2	17	27	2.7
DLiPC:DPPC	CHOL	6 : 4 : 2	17	27	2.8
DLiPC:DPPC	CHOL:CHIM	6 : 4 : 1.6 : 0.4	17 (1:4)	27	2.9

Supplementary Table 7. Compositions, trajectory lengths and temperatures of simulations of mixtures with the phospholipids DPPC or DPPC and POPS and one of or a mixture of CHIM-LX and CHOL.

phospholipid	compound	sterol content (mol%)	temperatures (°C, 10 °C steps)	length (ns)
DPPC	CHIM-LX	30	27-67	600
DPPC	CHIM-LX/CHOL (1:1)	30	27-67	600
DPPC	CHIM-LX/CHOL (1:4)	30	27-67	600
DPPC:POPS (2:1)	CHIM-LX	20	27-67	600
DPPC:POPS (2:1)	CHOL:CHIM-LX (1:4)	20	27-67	600

Supplementary References

1. A. K. Smith, J. W. Wilkerson, T. A. Knotts, Parameterization of Unnatural Amino Acids with Azido and Alkynyl R-Groups for Use in Molecular Simulations. *J. Phys. Chem* **2020**, 6253 (2020).

2. P. Drücker, M. Pejic, D. Grill, H.-J. Galla, V. Gerke, Cooperative Binding of Annexin A2 to Cholesterol- and Phosphatidylinositol-4,5-Bisphosphate-Containing Bilayers. *Biophysical Journal* **107**, 2070–2081 (2014).
