# Supplementary material for: CHIMs are versatile cholesterol analogs mimicking and visualizing cholesterol behavior in lipid bilayers and cells
Source: Commun Biol. 2021 Jun 11;4:720. doi: 10.1038/s42003-021-02252-5 (PMC8196198; doi:10.1038/s42003-021-02252-5)
Supplement: Supplementary file 2 — Description of Supplementary Files [file 42003_2021_2252_MOESM2_ESM.pdf]

Description of Additional Supplementary Files

File name: Supplementary Data 1

Description: Underlying data for charts and tables.
